# Associating transcription factors to single-cell trajectories with DREAMIT

**DOI:** 10.1186/s13059-024-03368-7

**Published:** 2024-08-14

**Authors:** Nathan D. Maulding, Lucas Seninge, Joshua M. Stuart

**Affiliations:** UCSC Genomics Institute, Biomolecular Engineering, University of California, Santa Cruz, USA

## Abstract

Inferring gene regulatory networks from single-cell RNA-sequencing trajectories has been an active area of research yet methods are still needed to identify regulators governing cell transitions. We developed DREAMIT (Dynamic Regulation of Expression Across Modules in Inferred Trajectories) to annotate transcription-factor activity along single-cell trajectory branches, using ensembles of relations to target genes. Using a benchmark representing several different tissues, as well as external validation with ATAC-Seq and Perturb-Seq data on hematopoietic cells, the method was found to have higher tissue-specific sensitivity and specificity over competing approaches.

**Supplementary Information:**

The online version contains supplementary material available at 10.1186/s13059-024-03368-7.

## Background

A cell’s type and state are a product of gene regulatory mechanisms controlled by transcription factors. Transcription factors (TFs) play a central role in governing the transitions between different cellular states. These transitions are driven by the dynamic regulation of gene expression orchestrated by TFs. TFs bind to specific genomic regions, exerting precise control over the activation or repression of target genes. This regulation is critical for processes such as cellular differentiation, development, and responses to environmental cues. The identification of TFs responsible for orchestrating these transitions is a fundamental endeavor in understanding the molecular basis of cell state dynamics.

**Fig. 1:**
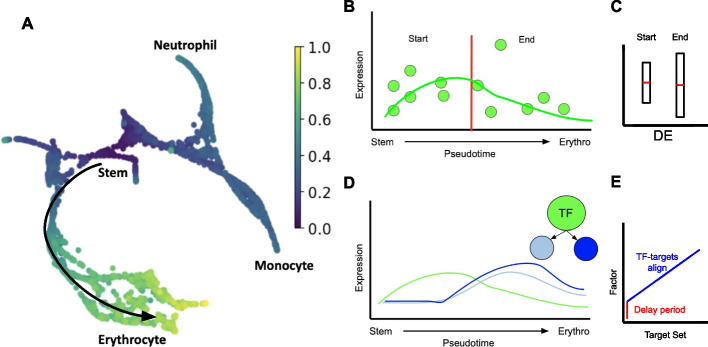
Associating transcription factors (TFs) to trajectory branches via identification of TF-to-target coexpression along pseudotime. **A** Expression across cell transitions in trajectory branches is used by DREAMIT to infer a dynamic views of TF-to-target gene regulation. **B** Expression levels of a hypothetical gene in individual cells (*y*-axis) illustrating the division into arbitrary “start” and “end” states along the pseudotime of a theoretical differentiation process from stem cells to differentiated erythrocytes (*x*-axis). **C** Differences in the expression level between “start” and “end” states may not exist which may cause Differential Expression (“DE”) approaches to miss other patterns in the data (e.g., concordant fluctuations in the middle of pseudotime). **D** DREAMIT models the entirety of the expression on the branch and assesses TF-to-target relationships that look for a consistent relationship between the expression levels (*y*-axis) of a TF (green line) and its target genes (blue lines) along pseudotime (*x*-axis). **E** Alignment plot showing one TF (*y*-axis) aligned to a “typical” target from a target set (*x*-axis) illustrating how allowing for a lag or delay (red line) can help a metric pick up on an association between a TF and its targets over a subset of pseudotime (blue line) in which all the targets have the same lag in expression relative to the TF

In recent years, advances in single-cell sequencing and transcriptomic methods have granted researchers the ability to scrutinize gene regulatory networks with great sensitivity and specificity. Moreover, the emergence of “cell trajectory” inference methods has enabled the identification of transitions between different cell states [1, 2]. Trajectory methods identify changes in development, maturation, or response to environmental queues using gene expression changes across cells having similar transcriptomes. The dynamic regulation of genes can then be inferred by following their relative expression across cells along a trajectory “branch” transitioning from one cell state to another (Fig. 1A).

Unraveling dynamic transcription factor regulation of cell states from single-cell RNA sequencing (scRNAseq) data presents several challenges. scRNAseq data itself introduces analysis complications, mainly due to its vast scale and sparsity [3]. These include issues like gene expression dropouts, stochastic variations, delayed target responses, and disparities in chromatin accessibility. These factors can result in inconsistencies in known factor-to-target regulations [4–6].

To address these issues, the first approaches to infer Gene Regulatory Networks from scRNAseq data used “pseudobulked” transformations in which cell expression was grouped across related cells. Most recently, a growing number of methods are available to infer GRNs specifically from scRNAseq data (see [3, 7] for good reviews). To complement the information in single-cell RNAseq data, some approaches extend to include multi-omic data, for example, the addition of ATACseq measured chromatin accessibility during the construction of GRNs [3]. Nevertheless, multi-omic data is both costly and not yet widely accessible. Thus, methods that extract TF-target relationships with strong support only from single-cell RNAseq data are still needed.

Importantly for this study, given a GRN, few methods exist to annotate which particular TFs are relevant for a particular context of the data. For example, even if a GRN is elucidated from a single cell dataset, it remains unclear which particular regulators to implicate as relevant for a cell state or cell transition residing in the dataset. As an analogy to bulk RNAseq analysis, GRNs can be inferred using methods like WGCNA [8] or ARACNe [9]. However, an additional step beyond GRN inference is needed to predict activities of genetic regulators using methods like MARINa [10], SPIA [11], and PARADIGM [12]. In the same way, approaches are needed to infer how genes within a GRN contribute to particular trends in a single-cell dataset. Current approaches use methods based on differential expression analysis, clustering, cell-type annotation, and dimensionality reduction [13–15]. Specific methods like TRADE-Seq [16] and PseudotimeDE [17] enable users to investigate differential gene expression as cells transition along a trajectory (see Fig. 1B–C). Meanwhile, approaches like SINGE [18] employ Granger causality ensembles to infer potential regulatory interactions. Nevertheless, methods tailored for the specific inference of TF activity along trajectories remain poorly studied and the performance of methods remains a challenging task, often lacking objective criteria for evaluation [7].

We introduce a novel method for implicating TFs to cell trajectories called DREAMIT—“Dynamic Regulation of Expression Across Modules in Inferred Trajectories.” DREAMIT aims to analyze dynamic regulatory patterns along trajectory branches, implicating transcription factors (TFs) involved in cell state transitions within scRNAseq datasets (see Fig. 1D–E). DREAMIT uses pseudotime ordering within a robust subrange of a trajectory branch (*pseudotime focusing*) to group individual cells into bins. It aggregates the cell-based expression data into a set of robust pseudobulk measurements containing gene expression averaged within bins of neighboring cells. It then smooths trends after searching for an optimal fitting spline across the bins (see the “Methods” section). DREAMIT rejects further analyzing branches that produce highly variable smoothing estimates (covariation in spline fitting parameters found to be greater than 1.0 across 80% subsampling; see the “Methods” section) as these branches may represent sparse or noisy parts of the data that could produce unreliable TF inferences.

Using the transformed smoothed data, it calculates the association between a TF and all of its predicted targets according to the TRRUST database assessed using multiple metrics (e.g., Pearson correlation, Mutual Information, and Dynamic Time Warping distance). Finally, DREAMIT uses a Relational Set Enrichment Analysis (RSEA) test to evaluate the significance of the TF-to-target associations and identify a core set of targets (*target focusing*) compared to a background model, which consists of arbitrarily selected targets.

We evaluated the performance of DREAMIT by measuring its ability to recover TFs with known relevance to several datasets. Our evaluation, although serving as a “bronze standard,” is based on the lack of suitable reference datasets with known underlying regulations driving the major differences among cells, as previously noted [18]. To assess DREAMIT’s performance, we employed a TF-Marker database, which allowed us to determine its effectiveness in identifying TFs known to play essential roles in specific tissues, previously established as high-confidence markers for those tissues [19]. While the evidence from gene expression alone should be viewed with caution as coexpression of transcription factors to targets is correlative and not causative, our findings revealed that DREAMIT outperformed traditional approaches in associating tissue-relevant TFs, including differential expression analysis and GENIE3, in several instances.

## Results

### DREAMIT identifies distinct PBMC markers

**Fig. 2:**
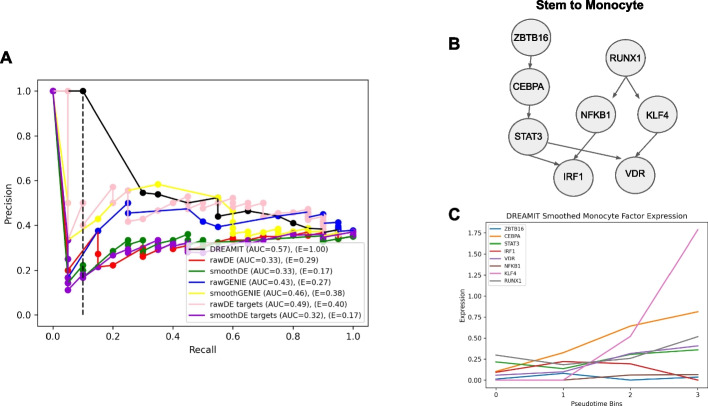
Performance of inferring blood differentiation TFs on the PBMC dataset. **A** Precision-recall curve measuring rate of identifying blood-related transcription factors annotated in TF-Marker DB from the analysis of the PBMC dataset. A dotted line is plotted on the precision-recall to denote where early-precision is considered. **B** TF-TF relationships found by DREAMIT depicted for the stem to monocyte branch from this PBMC dataset. **C** The expression (*y*-axis) of the TFs from part B plotted across pseudotime (*x*-axis)

To evaluate DREAMIT’s specificity in a highly curated setting in which confident tissue-specific transcription factor (TF) regulation is well known, we chose the blood marrow dataset from Paul et al. [20]. This dataset contains stem cells transitioning to various blood cell types (erythrocytes, monocytes, neutrophils) suitable for trajectory branch inference and contains a well-characterized set of marker genes. We estimated the accuracy of the methods using an average of 16.3 markers per branch by calculating precision-recall, and early precision (see the “Methods” section). DREAMIT was found to have the highest average precision and early precision (AUC = 0.57, *E* = 1.00) while the other approaches had lower estimates—smoothGENIE (AUC = 0.46, *E* = 0.38), rawGENIE (AUC = 0.43, *E* = 0.27), rawDE (AUC = 0.33, *E* = 0.29), smoothDE (AUC = 0.33, *E* = 0.17), rawDEtargets (AUC = 0.49, *E* = 0.40), and smoothDEtargets (AUC = 0.32, *E* = 0.17) (Fig. 2A). These estimates demonstrate that DREAMIT on average achieves levels of precision moderately higher than chance expectation as 35% of the transcription factors were tissue-related for this analysis. For example, the method achieves both a precision and recall of 0.55 which is significantly different than chance guessing at the 0.05 level (*P* < 0.026, hypergeometric test). In addition to tissue specificity, we also compared DREAMIT to Perturb-Seq results in hematopoiesis from Lara-Astiaso et al. [21].

Eight TFs in common were either tested by Perturb-Seq (i.e. one of the 81 TFs chosen as relevant to the Lara-Astisao et al. hematopoietic study) and that had reliable target sets and data amenable for DREAMIT analysis on the PBMC data. For example, 37 of the Perturb-Seq 81 overlapped with the TRRUST database, and, out of these, 23 were retained after scanpy filtering on the PBMC dataset. Eight of these 23 could be tested with DREAMIT (whereas 15 could not due to having target set sizes that were too small after eliminating those targets that also passed scanpy filtering). The TFs annotated to either the monocyte or erythrocyte branches had a high degree of overlap with Perturb-Seq data that report on factors that modulate these lineages (6 out of 8 TFs) and the downstream differential expression observed in marker genes had a higher concordance with those TFs selected by DREAMIT compared to the two that were not selected (Additional file 1: Fig. S1). The two TFs selected as non-relevant by DREAMIT indeed had lower Perturb-Seq scores across all of the lineages. While these results are consistent with DREAMIT identifying relevant TFs, the small number of TFs in common makes it impossible to calculate a quantitative level of specificity.

### DREAMIT inference of gene regulatory logic for PBMC fate specification

The majority of transcription factors (TFs) identified by DREAMIT were annotated as markers of stem cells or blood cells, which aligns with the input data from the Paul et al. dataset. This dataset represents the transition from stem cells to differentiated blood cell types. For instance, on the trajectory from stem cells to erythrocytes, DREAMIT found 16 significant TFs. Among these, five were well-known blood and peripheral blood markers based on the TF-marker database (RUNX1, GATA1, EGR1, STAT1, ETS1). Furthermore, three other TFs were stem cell markers (YBX1, MYC, RELA). Out of the remaining eight TFs identified by DREAMIT, six had established literature support connecting them to roles in stem cell and peripheral blood mononucleocyte (PBMC) development, such as DNMT1 [15], EZH2 [16, 17], E2F4 [18, 19], KLF6 [20, 21], NFE2L2 [22], and TP53 [23, 24]. The last two TFs, MYB and MYCN, had a less clear relationship (Additional File: Table S1).

On the trajectory from stem cells to monocytes, DREAMIT identified a total of 13 significant TFs. Six of them (CEBPA, ETS1, IRF1, ATF4, RUNX1, STAT3) were blood and peripheral blood markers, while three were recognized as stem cell markers (IRF8, KLF4, NFKB1). Among the remaining four TFs on this trajectory (ZBTB16, MYCN, ELF1, VDR), only the vitamin D receptor (VDR) had established literature supporting its involvement in monocytes [25, 26]. Perturb-seq findings here (see Additional file 1: Fig. S1).

To further investigate how DREAMIT findings provide insight, a TF-to-TF network was created alongside their temporal order of activation. For the significant DREAMIT findings from the high fidelity Paul et al. PBMC dataset [20], TF-to-TF relationships were plotted for the stem-to-monocyte trajectory (Fig. 2B). TFs are connected in the network if they were recorded as linked in the TRRUST database and significant by DREAMIT. Analyzing the (smoothed) expression patterns of TFs in the network revealed a correlation with the temporal changes in expression and the connectivity in the TF-to-TF network, as illustrated in Fig. 2C. For example, the factors STAT3 and RUNX1 are upregulated at the initial time point and their targeted TFs are upregulated at a later time point, consistent with the network inferred using the TRRUST TF-to-TF relationships [22–24]. In another example, a concurrent increase in ZBTB16 and CEBPA (regulated by ZBTB16) was observed followed by increases in their downstream target TFs—STAT3 (regulated by CEBPA) and VDR (regulated by STAT3). On the stem-to-erythrocyte trajectory branch, DREAMIT identified an analogous TF-to-TF network (see Additional file 1: Fig. S2). RUNX1, EGR1, MYCN, and ETS1 increased in the early stages of the trajectory branch with widespread increased expression across the TF network in later stages. GATA1 and DNMT1, both regulated by a single distinct TF, show increases in expression following increases in their single regulator (MYC → GATA1, DNMT1 → TP53).

### DREAMIT Identifies tissue-relevant TFs at a higher rate than standard approaches

Systematic assessment of a method’s accuracy in identifying TFs for a specific trajectory branch requires access to a diverse range of tissues with well-documented TF roles. Unfortunately, for many tissues, comprehensive single-cell analyses have not yet been conducted to establish a reliable set of TFs. Nonetheless, some relevant information has been gathered and stored in repositories like TF-Marker DB [13]. To address this challenge, we gathered seven datasets [25] in which at least one TF was found in both TRRUST and TF-Marker DB and was annotated as a regulator in tissues examined in the experiments. In total, this “bronze standard” benchmark encompassed 84 TFs identified as pertinent, resulting in 207 instances of TF associations with various datasets. This benchmark covered 15 trajectory branches across six different tissues, including the brain, heart, embryo, retina, bone marrow, and testis.

To assess the methods’ capacity to identify tissue-specific TFs in the benchmark datasets, we employed a precision-recall analysis. This approach is suitable for situations where we anticipate a far larger number of negatives than positives, primarily because we treat all unknown markers in the benchmark as negatives. To facilitate a robust comparison, we evaluated the overall performance of DREAMIT, Differential Expression (DE), and GENIE3 across all trajectory branches and tissues. To gauge the performance of these methods, we used the area under the curve (AUC) to evaluate their general performance across all recall levels. Additionally, we used “early precision” (E) [12] to assess performance under a strict confidence threshold, where only the top-ranked relationships are taken into account for calculation.

**Fig. 3:**
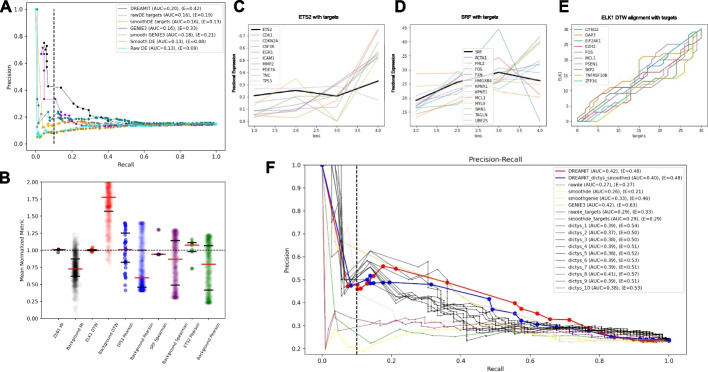
Performance inferring tissue-specific TFs from single cell benchmark datasets. **A** Precision (*y*-axis) versus recall (*x*-axis) measuring each method’s ability to detect TFs from one of the 15 benchmark trajectory branches in which true positive TFs were assumed to be those annotated by TF-Marker DB as previously associated with the tissue assayed by the experiment. **B** DREAMIT compares the TF-target relationship distribution to a background for each constituent metric and reports significant TFs through a Kolmogorov–Smirnov test. Examples of five different TFs with different metrics plotted along the *x*-axis (colors indicate TFs); each TF plotted as a pair with the left side showing the observed metric for the targets of the factor and the right side showing the distribution of randomly selected targets. **C** Illustration of ETS2 factor with its targets found to be significant by Pearson. To visualize genes of different expression scales in one plot, each gene’s expression from one bin was divided by that gene’s expression summed across all bins (fractional expression; *y*-axis). **D** Illustration of SRF found to be significantly correlated with its targets using Spearman correlation. Fractional expression (*y*-axis) was used for visualization purposes. **E** Dynamic time warping picked up on a significant relationship between ELK1 (*y*-axis) and its targets (*x*-axis); the alignment graph illustrates that the targets all maintain a relationship with the factor but there is variability from one target to the next in terms of the exact nature of the relationship. **F** DREAMIT and alternate methods’ precision-recall of tissue specificity based on the TF-Marker database evaluated on, and aggregated over, three different branches—B-cell, monocyte, and erythroid lineages. Results for several methods were compared including: Dictys (10 black lines), DREAMIT using raw, unprocessed data and its own spline smoothing (red), DREAMIT using pre-processed and smoothed data from Dictys (blue), Genie3 using raw data (orange) and Dictys-smoothed processed data (pink), as well as other methods and preprocessing (see legend). The early precision AUC is taken at 0.1 recall (dashed vertical line)

DREAMIT had the highest average precision (AUC = 0.20) surpassing its competitors, rawDE (AUC = 0.13), smoothDE (AUC = 0.13), rawDEtargets (AUC = 0.16), smoothDEtargets (AUC = 0.16), rawGENIE (AUC = 0.16), and smoothGENIE (AUC = 0.18) (Fig. 3A). Additionally, DREAMIT demonstrates a much stronger early precision (*E* = 0.42) compared to rawDE (*E* = 0.09), smoothDE (*E* = 0.08), rawDEtargets (*E* = 0.19), smoothDEtargets (*E* = 0.13), rawGENIE (*E* = 0.33), and smoothGENIE (*E* = 0.21).

The individual metrics of DREAMIT (excluded from Fig. 3A for clarity) maintained their respective specificity rankings when assessed by precision-recall with the exception of MI (AUC = 0.17) and Rolling (AUC = 0.18), which both fall behind Pearson (AUC = 0.22), Spearman (AUC = 0.21), and DTW (AUC = 0.19). On the other hand, the early precision of these component methods is ranked quite differently with Rolling (*E* = 0.43) scoring the best (even slightly superior to the DREAMIT ensemble, *E* = 0.42) followed by MI (*E* = 0.38), DTW (*E* = 0.35), Spearman (*E* = 0.28), and Pearson (*E* = 0.28), respectively. Both the component methods and the ENSEMBLE outperformed DE and GENIE3 in average and early precision.

To further compare DREAMIT, DE, and GENIE3, we created an upset plot to view the distinct and common TF-to-branch association pairs found across all of the 15 branches in the benchmark (Additional file 1: Fig. S3A). The tissue-specific TF-to-branch predictions found at FDR < 0.05 in each method are shown. DREAMIT finds the most number of TF-to-branch associations (109; 7.3 TFs per branch) followed by rawGENIE (58; 3.9 TFs per branch), rawDE (40; 2.7 TFs per branch), rawDEtargets (25; 1.7 TFs per branch), smoothGENIE (18; 1.2 TFs per branch), smoothDEtargets (17; 1.1 TFs per branch) and smoothDE (10; 0.7 TFs per branch). DREAMIT produced the largest number of TF predictions per trajectory branch making it the most sensitive method. In addition, DREAMIT shares the most overlap with rawGENIE, 52 associations (89.6%), and with rawDE, 33 associations (82.5%). We also investigated the overlaps of TF-to-branch associations found by the components of DREAMIT (data not shown). DTW found the most associations (73) followed by MI (71), Rolling (66), Pearson (64), and Spearman (39). There was a high degree of overlap among all of the methods. Spearman had the least overlapping associations, but 100% of its findings were also reported in one of the other 4 methods. Mutual information and DTW had the most exclusive TF-to-branch associations. Overall 16.5% of the associations found by DREAMIT were found by all component methods, and 66.2% were found by at least two.

Taken together, the vast majority of tissue-specific TF-to-branch associations found by the two competing methods were also found by DREAMIT, and in addition, DREAMIT found more than 50 associations missed by these methods. The total number of associations that could have been reported in this analysis was 130. This means that DREAMIT found 83.8% of tissue-specific associations, while rawGENIE and rawDE found 44.6% and 30.8%, respectively. DREAMIT had the highest degree of specificity and the highest percentage of tissue-specific markers.

DREAMIT found several cases, missed by other methods, in which the TF-to-target distribution was distinct from the background and found to be significant with a KS test (Fig. 3B). First, the marker ETS2 (Fig. 3C) was found to be significant according to the DREAMIT Pearson method (Rsq = 0.77, FDR < 0.001), as well as the marker SRF (Fig. 3D) on the same cardiac trajectory branch by the DREAMIT Spearman method (Ssq = 0.70, FDR < 0.005) [26]. DREAMIT captured these two annotated, biological markers in the trajectory data, while all other methods overlooked this as significant with the exception of rawDEtargets for ETS2 (pval < 0.005). Second, the marker TP53 (data not shown) was observed to be significant by DREAMIT Pearson (Rsq = 0.77, FDR < 1e − 7) [25], despite the large number of targets (*n* = 78) introducing potential noise into the calculation of the Pearson correlation. This demonstrates that DREAMIT is able to find strong TF-to-target relationships in both small and large target sets. This finding was missed by rawDE, rawDEtargets, smoothDE, and smoothGENIE, but was reported to be significant by rawGENIE and smoothDEtargets.

The DREAMIT DTW method also detected a significant association for ELK1 (*D* = 0.24, FDR < 0.01) (Fig. 3E), an association that was missed by all other methods [26]. Finally, the DREAMIT MI method found significance in the ZEB1 marker (MI = 2.35, FDR < 0.005), which was missed by rawDE, smoothDE, rawDEtargets, smoothDEtargets, and rawGENIE, but was found by smoothGENIE, suggesting that the smoothing spline implemented provides benefit to other methods of analysis.

To demonstrate the significance and minimal variability of the above results, their distributions were plotted as a swarm plot. The markers ZEB1, TP53, SRF, and ETS2 were all significantly above the background indicating a strong relationship found through either MI content, Pearson correlation, or Spearman correlation. The marker ELK1 was significantly below the background DTW distance, which indicates a stronger DTW alignment for ELK1. Altogether, these findings demonstrate that DREAMIT associates TFs to trajectories consistent with their known tissue specificity and that these findings are missed by DE and GENIE methods in many cases.

To expand the evaluation of DREAMIT to a more comprehensive independent dataset, we compared the performance of DREAMIT and the other methods to a recent multi-omic study called Dictys [27], which used a probabilistic model to incorporate ATACseq and RNAseq for TF activity inference. All methods were compared based on their performance to Dictys on the Dictys’ human hematopoietic dataset analyzed by the Dictys authors with their published trajectory solution (using their STEAM method [28]). Surprisingly, we found that the performance of DREAMIT and Genie3 were comparable to Dictys (DREAMIT and Genie3 both achieved AUCs of 0.42, while Dictys achieved 0.41 overall), with Genie3 having slightly higher early precision and DREAMIT higher late precision (see Fig. 3F). Notably, DREAMIT’s and Genie’’s precision was higher than Dictys over most recall levels considering all 10 different temporal GRNs produced by Dictys for each of the three trajectory branches. The alternate methods were not as competitive. On some of the branches, DREAMIT had superior performance. On the B-cell branch for example, DREAMIT had a better overall performance (0.49) compared to the best precision achieved by Dictys (0.41) or Genie (0.45) (see Additional file 1: Fig. S3B–D for a breakdown of results on all three hematopoietic branches). We found that using the Dictys smoothing of the data did not influence the performance of the methods.

## Discussion

In this work, we presented DREAMIT, Dynamic Regulation of Expression Across Modules in Inferred Trajectories, a novel framework for testing and identifying dynamic gene regulation in TF to target relationships. The method can be used downstream of any method that projects single-cell data into a lower dimensional manifold and derives a cell trajectory solution (e.g., Monocle instead of Slingshot used in this study). The method was developed to aid researchers in identifying the gene–gene regulatory relationships governing the state transitions cells undergo detectable in associations between their transcriptomes. Previous methods either ignore gene regulation, such as TRADE-seq or PseudotimeDE [17], or were not designed specifically for single-cell trajectories, such as GENIE3 [29]. As such, DREAMIT provides a complementary perspective on interpreting gene regulatory mechanisms from scRNAseq data.

TF-target sets, whether taken from databases or user-supplied, will likely contain targets that are irrelevant for the analysis of a particular tissue and may not reliably detect an association of a TF to a trajectory branch. For this reason, we introduced the use of several association metrics (e.g., Pearson, Mutual Information, Dynamic Time Warp, etc.) as well as the use of a Relational Set Enrichment Analysis (RSEA) to detect the significance of a target set’s association relative to a random background that can tolerate such noise in the target set. Furthermore, we found that using a target focusing step, much like an enrichment analysis can identify a “leading edge” of contributing pathway genes to a differential expression signature, helps boost the RSEA detection signal.

In a benchmark set of data encompassing six tissues, we found that DREAMIT exceeded the performance of Differential Expression (DE) and GENIE3. DREAMIT had both a higher sensitivity overall and captured more tissue-specific markers. In summary, DREAMIT was shown to have higher sensitivity, finding over 80% of the tested markers, with higher ROC, precision-recall, and early precision compared to DE and GENIE3-based methods.

In the PBMC dataset, DREAMIT also had the highest specificity in terms of ROC (AUC = 0.66), precision-recall (AUC = 0.57), and early precision (*E* = 1.00). On the erythrocyte branch, DREAMIT found 16 significant TFs, 5 of which were known blood markers and 3 were known stem cell markers. On the monocyte branch, there were 13 significant TFs, 6 were known blood markers and 3 were stem cell markers. Of the significant TFs found by DREAMIT that were not established markers in the TF-marker database [19], 9 of them had established or emerging roles in stem and PBMC development in the literature (see Additional file 1: Table S1). For example, the finding association of VDR with monocytes was not documented in the TF marker database but has been demonstrated in recent literature [30, 31]. Thus, among DREAMIT’s predictions are potential new examples of tissue-specific regulation by novel factors.

Additionally, DREAMIT was able to find 26 TF-to-branch associations missed by any other competing method (Additional file 1: Fig. S3A). For example, significant marker findings of ETS2, SRF, TP53, ELK1, and ZEB1 were captured by DREAMIT, but missed by the others. DREAMIT presumably can pick up on these overlooked cases because it considers multiple relationship modalities between TFs and their targets (e.g., Pearson, DTW, and MI).

DREAMIT uses only scRNAseq data to implicate TFs to particular cell transitions. As such, the association of TFs to branches based on the gene coexpression of targets should be viewed as suggestive. In addition, since the metrics are based on coexpression relations and since the directionality of pseudotime may not reflect “real time” in a cell, the TF could promote or repress activity along a branch. As new multi-modal datasets are increasingly published (e.g., ATACseq and RNAseq on the same cells), an obvious question becomes whether incorporating additional datasets would improve the performance of a TF association method like DREAMIT. Our results of comparing DREAMIT to Dictys, which used expression together with chromatin accessibility information while DREAMIT used only expression revealed that DREAMIT identified as many tissue-specific TFs (and sometimes slightly more at the early precision levels) compared to Dictys and that many of the TFs were those reported by the authors. The recent PerturbSeq study in mouse by Lara-Astiaso et al. [21] in which TFs were systematically knocked out and then specific marker gene expression assayed, also showed a high concordance with DREAMIT findings from the Paul et al. dataset [20]. We found that, out of the 8 murine TFs reported for the HSC-to-monocyte or HSC-to-erythrocyte lineages, six of the human orthologs had been associated by DREAMIT to either one of these branches. Furthermore, the two that had not been associated with either branch showed much lower levels of marker gene differential expression in the monocyte and erythrocyte branches when the TFs were knocked out (see Additional file 1: Fig. S1). Taken together, the Dictys and Perturb-Seq comparisons further demonstrate the advantage of DREAMIT to compensate for the lack of multi-omic data through the use of TRRUST relationships and RSEA.

One limitation of DREAMIT is that it considers one TF at a time even though it is known that TFs work together in combination. Even so, several significant associations are found by considering individual TFs in isolation. Extensions of the work are possible that could test combinations of TFs. For example, starting with pairwise TF combination detection, one could test the association between two TFs as well as all pairwise associations between the distinct members of their target sets using the same metrics and statistical tests defined in this work. The drawback of the approach would be the difficulty currently in evaluating its success as there is limited availability of datasets in which TF combinations have been annotated as relevant.

While DREAMIT exceeded competitors in our evaluations, all of the methods had low precision. This is likely due to the limited availability of relevant TFs associated to a particular tissue and to specific branches produced by trajectory inference. For example, using TFs from the TF Marker database allowed us to consider multiple datasets for the evaluation, but it assumed TFs annotated to a tissue were relevant for any/all branches in a dataset that assayed a specific tissue. It is certainly possible that other TFs or other biological differences underlie the variation in the observed transcriptomes of individual cells. There is clearly a need for well-annotated datasets that can be used as benchmarks for gene regulatory network inference in single-cell analyses [18]. As more datasets with multiple data modalities become available (e.g., ATACseq and RNAseq), it will be possible to develop sets of TFs relevant for branches in a more unbiased and systematic fashion.

DREAMIT code is available on GitHub https://github.com/nathanmaulding/DREAMIT.git. The grid search that DREAMIT uses to find reasonable parameters for spline fitting contributes the biggest impact to running time and grows linearly with the number of cells along a trajectory. For example, the smallest dataset in this study took 14.8 s and the largest took 189,704 s. A typical dataset, like the one taken from Paul et al. [20] and provided as an example in the code repository, takes 1 h with 20 threads. Parallelization of the grid search to utilize nodes on a large compute cluster greatly improves running time as each parameter combination can be run independently.

## Conclusions

We developed and evaluated DREAMIT, a novel framework for investigating dynamic gene regulation in TF-to-target relationships gleaned from cell trajectories inferred in single-cell RNAseq data. DREAMIT was found to outperform baseline approaches in 15 different trajectory branches in a benchmark dataset and the well-characterized PBMC dataset. DREAMIT detected the association of TFs to tissue-specific trajectories in several instances where the association was missed by other methods, demonstrating its variety of metrics may help it detect some of the dynamic interdependencies preserved in the pseudotime inference provided by the cell trajectory analysis. Future application to single-cell time-series datasets like Klein et al. [32], rather than inferences based on pseudotime as was done here, could also prove useful insights into GRN relationships. In conclusion, DREAMIT offers a complementary approach for shedding light on TF-to-TF networks that govern the temporal regulation assayed by emerging single-cell datasets.

## Methods

DREAMIT contains the following major steps: (1) pseudotime ordering to bin individual cells together, (2) averaging gene expression for each bin, (3) fitting a spline model to derive gene expression that smooths trends, (4) calculating the association between a TF and all of its predicted targets according to the TRRUST database using several different metrics, (5) identifying the significance of the set of targets using an empirically sampled null model and identifying a core set of the targets using an enrichment test, and (6) retaining any TFs with a significant concordance after multiple hypothesis correction. These different steps are described in the following sections.

### Datasets and dependencies

DREAMIT relies on RNA expression data and the allocation of cells to specific branches, including their positions along these branches, which are represented as “pseudotime.” These assignments are determined through the application of a cell trajectory inference method, as illustrated in Fig. 1A. For this work, slingshot and PAGA were used to infer trajectories and pseudotime [33, 34]. PAGA was used for the Paul et al. dataset [20] since that study published a set of cell clusters that could be used as input to the PAGA method. Slingshot was used for the benchmark datasets (described next). The selection of Slingshot was based on the absence of pre-existing clustering assignments in these benchmark datasets and its well-documented performance in systematic evaluations [1]. The analysis is dependent on pseudotime assignments estimated by trajectory methods. Errors introduced by a trajectory method can impact which gene regulatory relationships can be identified. DREAMIT is downstream of trajectory methods and is therefore subject to the same errors. To mitigate producing inferences based on potentially unreliable data and/or trajectory solutions, DREAMIT fits a series of splines to 80% subsampled data for each branch. It only considers for further analysis those branches for which the fitted spline coefficients have a coefficient of variation that is under 1.0.

For benchmarking, we collected 7 datasets from EBI representing a diversity of tissues (including brain, heart, embryo, retina, bone marrow, testis) [25], a heart development dataset [26], and a dataset of stem to PBMC lineage [16] for testing DREAMIT. Each of these datasets has undergone preprocessing through the standard scanpy pipeline [13].

Traditional methods compare different states on the branch to each other in terms of differential expression, between a “start” and an “end” state (Fig. 1B–C). DREAMIT takes a different approach in which the full set of expression values along pseudotime are used to infer a relationship between a Transcription Factor (TF) and its target. To do this, the method uses a set of predicted linkages between regulators and targets. For this study, we used both the human and mouse regulator-target predictions contained in the TRRUST database [35], which contains interactions mined from over 11,000 PubMed articles. To convert mouse regulogs to human, we used the orthology mapping published by the Mouse Genome Informatics (MGI) consortium [36]. These known regulatory datasets are provided with DREAMIT or the user can choose to include their own regulator-target interactions.

### Trajectory pre-processing: pseudotime focusing, expression quantizing, and spline smoothing

DREAMIT bases its analysis in first producing a smoothed representation of the data through psuedotime focusing and spline fitting. The use of splines to model scRNAseq data along a trajectory branch follows previous work [16, 17]. We selected spline models that incorporated both goodness-of-fit and robustness.

**Fig. 4:**
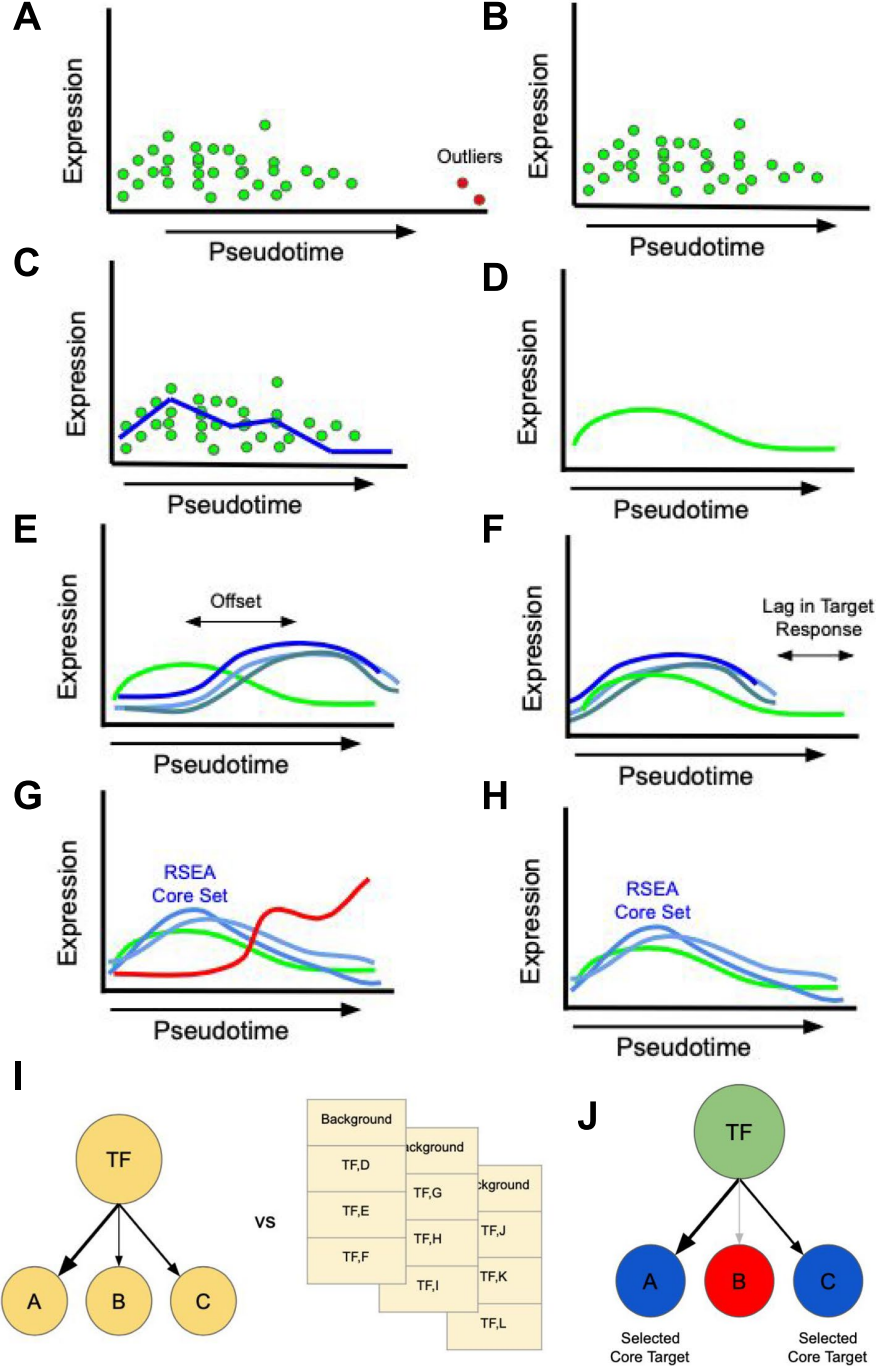
Detecting TF-to-target relations using pseudotime focusing, spline smoothing, target focusing, and significance assessment via random target selection.** A** Cells with outlier pseudotime assignments (red dots) compared to the other cells (green dots) are shown. **B** *Pseudotime focusing* removes outliers from the analysis and retains cells within 1.5 times the interquartile range of all pseudotime values of the branch (see the “Methods” section). **C** Cells are grouped into bins containing at least 10 cells per bin. The average expression of a gene is calculated from all the cells in a bin (blue line) and this bin-averaged expression is used for all subsequent analysis. **D** *Spline smoothing* incorporates information from cells in neighboring bins to further smooth out the expression changes in pseudotime (green curve). **E** DREAMIT quantifies TF-target relationships through pairwise tests of the spline-smoothed expression of the TF (green line) and its target genes (blue lines). **F** Illustration of a “rolling” metric incorporating pseudotime lag. A significant lagged correlation will be detected when several targets share the same delay. **G**. *Target focusing* employs Relational Set Enrichment Analysis (RSEA, see the “Methods” section) to identify a “core” set of targets with high association to the target (blue lines) while excluding the targets with weak or poor association (red curve). **H** 75% of the targets with the highest concordance to the factor are retained. **I** *Significance is assessed* by comparing TF-to-target metric scores to a random background in which random targets are chosen to be of the same size as the TFs original regulon (yellow lists) with a Kolmogorov–Smirnov test. **J** The core set of targets (blue nodes) found by RSEA are used in the statistical analysis

The assignments of cells to locations along a trajectory, commonly referred to as pseudotimes, can reflect a fairly irregular distribution in which the density of cells can vary appreciably from one area to the next. This irregularity in cell number along pseudotime could result in only a few cells, or even a single cell, having a disproportionately large influence in correlation or distance calculations made by DREAMIT (described below). For this reason, DREAMIT applies a *pseudotime focusing* step in which it retains the cells that have been assigned contiguous pseudotime values falling within 1.5 times the interquartile range of all pseudotime values of the branch (Fig. 4A–B). The outlier cells that could impact detecting robust trends across pseudotime are eliminated from all subsequent steps.

DREAMIT discretizes the assigned pseudotime values into discrete ordinal levels in order to buffer against further irregularities in the data. Discretization bins are chosen to have an equal number of pseudotime increments, where each bin also has a minimum of 10 cells. Expression levels are then averaged across all cells in the same bin(Fig. 4C). Thus, the result of the quantization step produces a type of pseudobulk dataset in which gene expression levels are averaged across cells landing in the same ordinal bin. DREAMIT attempts different resolutions of discretization by varying the number of bins from 4 to 100 and chooses a satisfactory number using a hyperparameter search step (described below). It also provides the option of using the raw expression data for the next step of spline smoothing rather than the binned pseudobulk data.

**Fig. 5:**
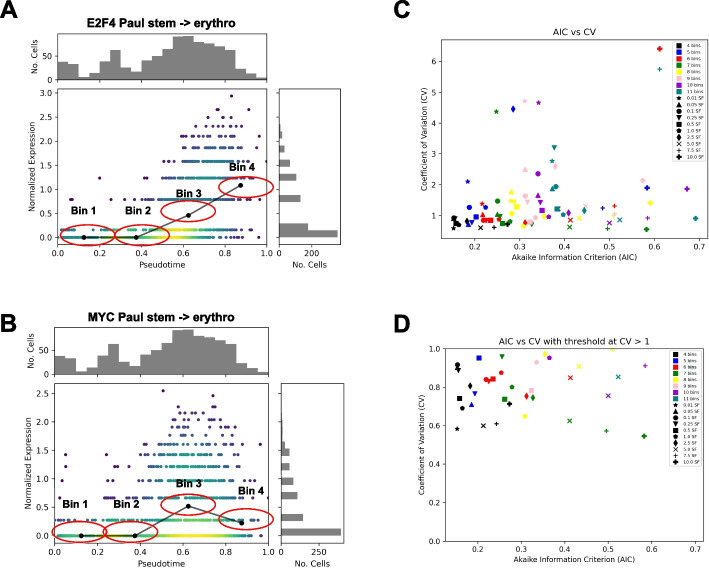
Modeling the gene expression across a trajectory. **A.** Illustration of E2F4 expression (y-axis) across pseudotime (*x*-axis) along the stem to erythrocyte trajectory branch from the PBMC dataset with normalized (Scanpy) expression (color indicates number of cells); spline-smoothed expression shown for each pseudtime bin (black line); red ellipses illustrate bin width. Histograms plot the distribution of cells at given expression increments (*y*-axis) and psuedotime increments (*x*-axis). **B.** Same plot as in A but for the MYC transcription factor. **C.** Each spline parameter choice produces a different fit to the data. Robustness of the fit was assessed by measuring the coefficient of variation (CV) across subsamples of the data (*y*-axis, see the “Methods” section). Goodness of the fit was assessed using Akaike information criterion (AIC) (*x*-axis). Parameter values that produce spline fits plotted toward the origin (bottom left) are preferred to those further away. **D** Same as in **C** but only showing parameterizations that achieve tolerable levels of robustness (CV > 1)

The expression levels of the retained cells could have substantial noise due to technical and biological factors; for example, noise due to the well-documented zero-inflated bias in single-cell RNAseq data [37] (see Fig. 5A–B for two examples of raw expression levels). In addition to only averaging the expression levels within a bin, DREAMIT uses a spline smoothing step to incorporate information from cells in neighboring bins (Fig. 4D). The discretized pseudotime units are treated as the independent variable and the average expression levels are fit with a zero-inflated negative binomial generalized additive model spline smoothing (NBGAMSS) of the expression values following the recent approaches of TRADE-Seq and PseudotimeDE [16, 17]. The number of cells in a bin is used as a “weight” for the fitted data point, placing more emphasis on areas with more support that are assumed to have more reliable estimates of average expression compared to areas with a fewer number of cells. A smoothing factor determines the number of resulting knots produced by the NBGAMSS (see Fig. 5A–B for two examples of smoothed expression). Note that other smoothing operations are possible such as the kernel smoothing used by SINGE [18].

The hyperparameters for a spline are selected by searching for a combination that maximizes the goodness and robustness of the fit, measured by Akaike information criterion (AIC) and coefficient of variation (CV), respectively. Subsamples are used to compute the coefficient of variation (CV) to reflect robustness. We subsample a trajectory branch by choosing 80% of the cells, without replacement, repeated 30 times. The average Akaike Information Criteria (AIC) is calculated across these subsamples to reflect the accuracy of the fit to the quantized data. Likewise, the CV is calculated across these subsamples. We search for a set of tolerable spline parameters—the number of bins and the smoothing factor—that minimize both the CV and the AIC. A perfect dataset would have an AIC and CV of 0. We used the distance to the origin of a spline’s associated AIC-CV pair as a measure of goodness that combines both the fit quality and robustness. To illustrate the spline selection process qualitatively, we plotted splines for ANKRD43 and FRY1 that had a good fit (low AIC and CV; Additional file 1: Fig. S4–5) as well as a poor fit (high AIC and CV; Additional file 1: Fig. S6–7). The differences in a poor modeling are evident at high bin numbers, but it should be noted that most spline fits are not extremely off base in terms of modeling, but the best parameters enhance the smoothness. All of the tested spline representations of the PMBC dataset [16] were plotted to assess their AIC (goodness of fit) and CV (robustness of fit)(Fig. 5C), with little to no trend being observed with the changing bin and smoothing factor parameters.

As RNAseq becomes more cost-effective, datasets will increase in resolution, decreasing the distance between cell state transitions, and the discretization step may not be necessary for some datasets. Thus, DREAMIT provides the option of using the raw expression data for spline smoothing without first performing the quantization binning step. We tested the influence of binning on the Paul et al. dataset and found that it produced TF predictions with more tissue specificity compared to predictions obtained without the binning step (see Additional file 1: Fig. S8a–b).

Expression data for each of the 100 most variable genes is used for the hyperparameter search. Once an optimal value for these parameters is found, the preprocessing is applied to all genes. In order to ensure that DREAMIT only ever reports on robust representations of the data the maximum threshold for coefficient of variation is set to 1 (Fig. 5D) in this and all other data analyses. Only trajectory branches that meet this CV criterion will be assessed by DREAMIT.

### Transcription factor to targets

To quantify relationships between a transcription factor (TF) and its targets, DREAMIT performs a series of pairwise tests (e.g., Pearson correlations or dynamic time warping alignments, see next section) between a particular TF and each of its predicted target genes. The targets of a particular TF are taken from the regulatory interactions recorded in the TRRUST database [35]. The number of targets for each TF in DREAMIT is a result of at least three factors: (1) which targets are linked to the TF in the TRRUST database, (2) are retained after scanpy filtering of the data to select for highly variable genes, and (3) are included after DREAMIT RSEA.

### DREAMIT metrics to detect a variety of TF-target relationships

The connection between the expression levels of a transcription factor (TF) and those of its target genes can vary from being straightforward and linear to more nuanced. This variation depends on the particular regulon (i.e., the strength of association between a TF and a large or small subset of its target genes). For that reason, DREAMIT includes several metrics to pick up on TF-target associations. For each TF with an associated target set, DREAMIT calculates Pearson correlation, Spearman correlation, Dynamic Time Warping Cost, and Mutual Information content [38–41]. We describe the details of the calculation of each of these methods in the following paragraphs.

Pearson correlation provides evidence regarding the strength of the linear relationship between the factor and its targets, while Spearman correlation reflects the strength of the monotonic relationship. Because the factor and targets can have positive or negative correlation, the metric must be squared to create a distribution that is comparable to a random background distribution. Therefore, it is primarily the strength of the relationship that is considered in DREAMIT, but all relationships are included in the report for the user to investigate.

Dynamic Time Warping (DTW) measures how well the expression pattern of a factor can be aligned to the pattern of a target. Target genes may follow a similar pattern as a regulator but have delayed timing that may be detectable in a single-cell dataset with enough resolution to reveal cellular processes. DTW finds an optimal match between the patterns that introduces the fewest delays in time [42]. Therefore, a factor with a small DTW distance from its targets implies that the targets are tightly controlled by the transcription factor with few delays in pseudotime.

Mutual Information (MI) measures the potentially non-linear dependency between the expression of a factor and that of a target gene. It measures how much a factor’s expression distribution reduces the uncertainty about the target’s expression distribution [43]. Including MI among the metrics expands the relationships that can be detected between TFs and targets but can produce results in which the nature of the regulatory interaction is not clear (e.g., in the cases where correlation-based metrics fail to pick up a relation).

In addition to the standard use of these metrics, a rolling metric calculation is also performed. In the rolling metric calculation, the possibility of a delay in targets responding to factor expression changes is considered by sliding the window in which the relationship is measured. In other words, a rolling window is applied to the target’s expression where each window interval is a bin of smoothed expression (Fig. 4E–F). Therefore, the rolling metric reports the bin increment delay where the peak strength of the delayed relationship occurs. DTW allows for delays in factor target relationships, but is flexible as to the synchronization. In contrast, the rolling metrics report significant factor target relationships with the same time delay, so that all targets respond to the factor expression at a similar pseudotime.

### Relational Set Enrichment Analysis (RSEA): detecting significant TF-target associations

In addition to a standard reporting of these prior known TF-target associations, DREAMIT uses a two-step Relational Set Enrichment Analysis (RSEA) to highlight the core set of relationships (Fig. 4G–J). RSEA begins by performing target focusing to capture the enriched target response followed by a Kolmogorov–Smirnov test (KS test) to assess significance. A transcription factor may activate a subset of its targets in a particular tissue. DREAMIT attempts to identify the set of utilized targets by identifying a focus set. Target focusing uses the provided set of targets for a factor (e.g., in this study, [35]). DREAMIT performs two steps to identify targets with consistent expression with the factor. First, it checks if the direction of regulation is consistent with the activation/inhibition annotation available from the TRRUST database. If the database has no annotation about the directionality of regulation (46% of relations in the TRRUST database), then this consistency check is skipped. Second, DREAMIT performs a focusing step to retain a set of targets that mutually have the strongest relations to the TF; i.e., keeps only those targets with the highest scores to the TF using the current choice of metric. A range of percentile thresholds was tested from 10 to 35 with similar results for 20 and above, with 20 and 25 having the best early precision (Additional file 1: Fig. S8c–d). The target focusing threshold is a hyperparameter of DREAMIT; 25 was used in this analysis. Targets with an association to the TF above the 25th percentile of a DREAMIT metric are retained in a core “target focus” set. Conversely, targets with deviant expression are filtered out in an effort to reduce false positives.

To determine the significance of a DREAMIT relationship metric for a TF, the distribution of metric levels between the TF and its targets is compared to a random background distribution. The background set is chosen to be equal to the largest target set considered. After the random targets are selected, the same metrics are calculated for the random set and then compared to the true factor-to-target distribution using the Kolmogorov–Smirnov test (KS test) [44]. A Benjamini–Hochberg correction [45] is then performed on the *p*-values generated for each factor by the KS test to account for false discoveries. Transcription factors with adjusted *p*-values < 0.05 are considered significantly non-random for the association in question. Both RSEA results and the full set of TF-targets are evaluated using this KS test and *p*-value correction method.

### Differential expression (DE)

DREAMIT uses a differential expression (DE) *t*-test between the start and end of a cell trajectory using the original data values (“Raw DE”) as well as using the spline-inferred data values (“Smooth DE”). The spline-inferred, Smooth DE approach uses a generalized additive model (GAM) as the basis for the DE test, similar to previous methods such as Monocle-2, TRADE-Seq, and PseudotimeDE [16, 17, 46]. For the DE test (either raw or smooth), DREAMIT divides a trajectory branch into two equal segments, the “start” and the “end.” For raw expression, outlier cells are pruned such that those with aberrant pseudotime values are ignored. If a gene shows a statistically significant change between these two conditions then it is considered differentially expressed. Statistics such as fold change, t-statistic, *p* value, and FDR are then reported for each gene on a branch for both the raw and smoothed data.

### Evaluating the specificity of TF to target relationships

To determine the specificity of DREAMIT, ROC, Precision-Recall, and early precision were assessed. True “hits'' are defined as transcription factors that are markers of the tissue under study. All other transcription factor findings are considered false hits. This assumption does not provide perfect accuracy as there are likely many factors that have yet to be classified in the TF-Marker database being used [19], and also every factor marker of a tissue need not be active at all times. However, this bronze standard metric does give an enhanced insight into DREAMIT’s ability to highlight tissue specificity. ROC, Precision-recall, and early precision were assessed branch by branch and as an aggregate. These specificity curves were determined for findings by Pearson correlation, Spearman correlation, dynamic time warping, mutual information, rolling calculations, and by the most significant method for each factor. By examining the TPR, FPR, precision, and recall under various *p*-value thresholds, the specificity of these methods can be assessed comparatively. For early precision, we followed previous works [18] that defined this metric as the precision at recall levels ≤ 0.1.

In a similar manner, competing approaches, differential expression (DE) and GENIE3 [29], were comparatively assessed against these DREAMIT methods. For DE, if the transcription factor (TF) is listed as a marker of the tissue under study then it is considered a true hit, while all other TFs are considered false hits. This is the same method used for DREAMIT. Additionally, the DE targets were assessed by comparing the T-statistics of the targets with a background set using the KS test. For the TF in question, it is considered significant if its targets score better than the random background. Both the scanpy processed expression (rawDE) and the spline smoothed expression from DREAMIT (smoothDE) were assessed for both TF DE and DE targets. For GENIE3, a distribution of weights is determined for TFs to known targets from TRRUST [35]. This is then statistically compared to a distribution of weights for TFs to randomly selected genes (representing the background) with a KS test. This is done for both scanpy processed expression (rawGENIE3) and the spline smoothed expression from DREAMIT (smoothGENIE3). Specificity is then assessed in the same way as DREAMIT where tissue markers are true hits and all other TFs are false hits.

We note that rawDE has some processing done (IQR pruning of cells with outlier pseudotime assignments). This processing was done, because often dividing a branch strictly on the original pseudotime assignments results in severe imbalances in the number of cells in the start and end segments. Therefore, some processing was still useful in this analysis above the traditional approach, even before results are considered.

### Evaluating the overlaps between DREAMIT and other methods

In order to compare the findings of DREAMIT, DE, and GENIE3 methods, a set of TF-branch pairs was recorded for each method’s predictions. For example, the pair (MEK, Branch-3) would record that MEK was associated with the third trajectory branch of the dataset. The TF-branch pair sets of each method were compared and visualized using an Upset plot (Fig. 3C–D). This was done for both the tissue-specific markers of the respective branches being assessed and for the non-markers for DREAMIT, rawDE, smoothDE, rawDEtargets, smoothDEtargets, rawGENIE, and smoothGENIE. Likewise, the DREAMIT subcomponent methods Pearson, Spearman, DTW, MI, and Rolling were also assessed via Upset plot for both tissue-specific TF marker and non-marker findings. We note that different upstream methods for manifold learning and trajectory inference could be used in conjunction with DREAMIT (such as scGNN [47], DESC [48], and scMGCA [49]).

### Evaluating specificity in reporting TF-markers in a high-fidelity PBMC dataset

To assess the specificity and biology of DREAMIT in a “silver standard,” a PBMC dataset was used [20]. Because this dataset was derived from mice, a set of regulogs [36] was used in this analysis so that downstream specificity and tissue markers can be assessed. Due to a higher proportion of TFs being categorized as blood-specific markers or not, the specificity for DREAMIT can more accurately be determined. ROC, precision-recall, and early precision are determined in the same way as above, using the TF-marker database [19]. Individual factors were then investigated further as to their status in the marker database and in the overall literature.

### Supplementary Information

Additional file 1. Supplementary figures

Additional file 2. Supplementary tables

Additional file 3. Review history

## Data Availability

All data used in this study came from publicly available external sources. The following scRNAseq datasets were used in the study with accession numbers: EBI’s Single Cell Expression Atlas (EBI) E-GEOD-134144 [50], EBI E-HCAD-13 [51], EBI E-HCAD-6 [52], EBI E-GEOD-130473 [53], EBI E-GEOD-98556 [54], EBI E-GEOD-75140 [55], ArrayExpress (AE) E-MTAB-7008 [56], AE E-MTAB-6268 [26], Gene Expression Omnibus (GEO) GSE139369 [27], GEO GSE72857 [20], GEO GSE60103 [21], GEO GSE124822 [21], GEO GSE213511 [21], and GEO GSE213506 [21], GSE213507 [21]. An open-source version of the DREAMIT source code is available on Zenodo at 10.5281/zenodo.13175583 [57] under the rights and permissions of the MIT open-source and OSI-compliant license: Permission is hereby granted, free of charge, to any person obtaining a copy of this software and associated documentation files (the “Software”), to deal in the Software without restriction, including without limitation the rights to use, copy, modify, merge, publish, distribute, sublicense, and/or sell copies of the Software, and to permit persons to whom the Software is furnished to do so, subject to the following conditions: The above copyright notice and this permission notice shall be included in all copies or substantial portions of the Software. THE SOFTWARE IS PROVIDED “AS IS”, WITHOUT WARRANTY OF ANY KIND, EXPRESS OR IMPLIED, INCLUDING BUT NOT LIMITED TO THE WARRANTIES OF MERCHANTABILITY, FITNESS FOR A PARTICULAR PURPOSE AND NONINFRINGEMENT. IN NO EVENT SHALL THE AUTHORS OR COPYRIGHT HOLDERS BE LIABLE FOR ANY CLAIM, DAMAGES OR OTHER LIABILITY, WHETHER IN AN ACTION OF CONTRACT, TORT OR OTHERWISE, ARISING FROM, OUT OF OR IN CONNECTION WITH THE SOFTWARE OR THE USE OR OTHER DEALINGS IN THE SOFTWARE.
