# Supplementary material for: Associating transcription factors to single-cell trajectories with DREAMIT
Source: Genome Biol. 2024 Aug 14;25:220. doi: 10.1186/s13059-024-03368-7 (PMC11323358; doi:10.1186/s13059-024-03368-7)
Supplement: Supplementary file 1 — Additional file 1. Supplementary figures [file 13059_2024_3368_MOESM1_ESM.pdf]

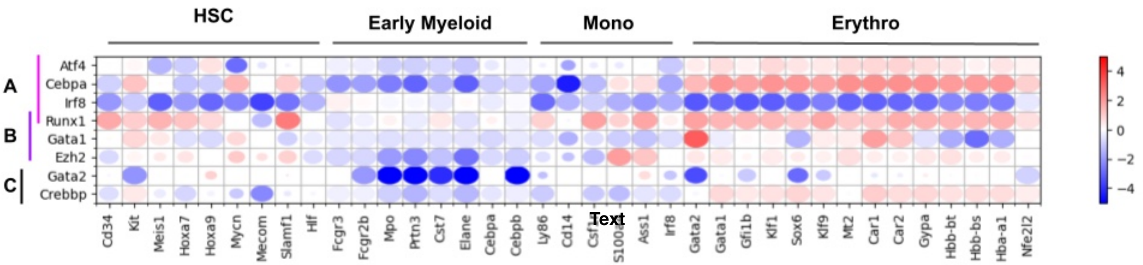

Fig S1. TFs scored by DREAMIT and the Perturb-seq experiment from Lara-Astiaso et. al. [21] were plotted against a set of markers for HSC, early myeloid, monocyte, and erythrocyte cell types. The color of each dot represents the logarithm base-2 of the fold change (a ceiling of -5 and +5 was implemented) and the size of a dot represents (1 - the adjusted P-value) reflecting the differential expression of a marker gene after a TF is knocked out. The fold change and P-values were obtained from the Lara-Astiaso et. al. publication. DREAMIT found TF set A (pink - Atf4, Cebpa, Irf8, Runx1) to be significant in the monocyte lineage, the TF set B (purple - Runx1, Gata1, Ezh2) to be significant in the erythrocyte lineage, and the TF set C (black - Gata2, Crebbp) to not be significant in either lineage.

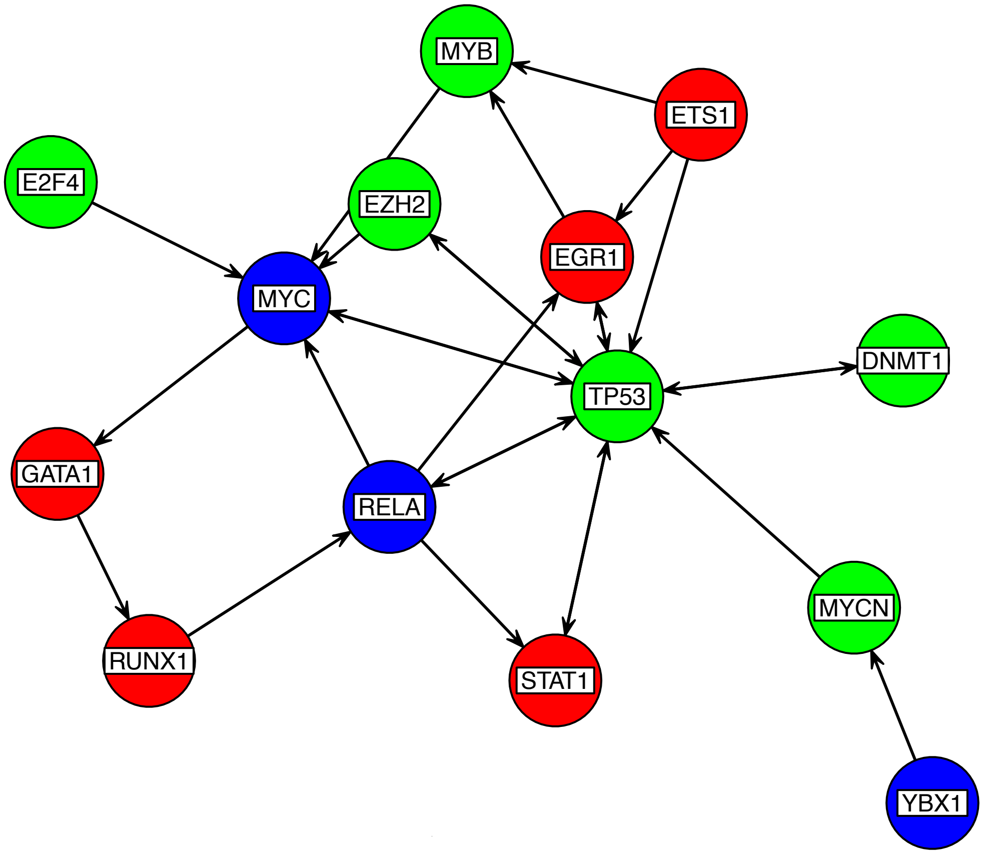

Fig S2. The TF-TF network displays directionality and regulation from TRRUST. The color of each TF node represents the cell type; red for blood marker, blue for stem marker, and green for others. These annotations were obtained from the TF-Marker database.

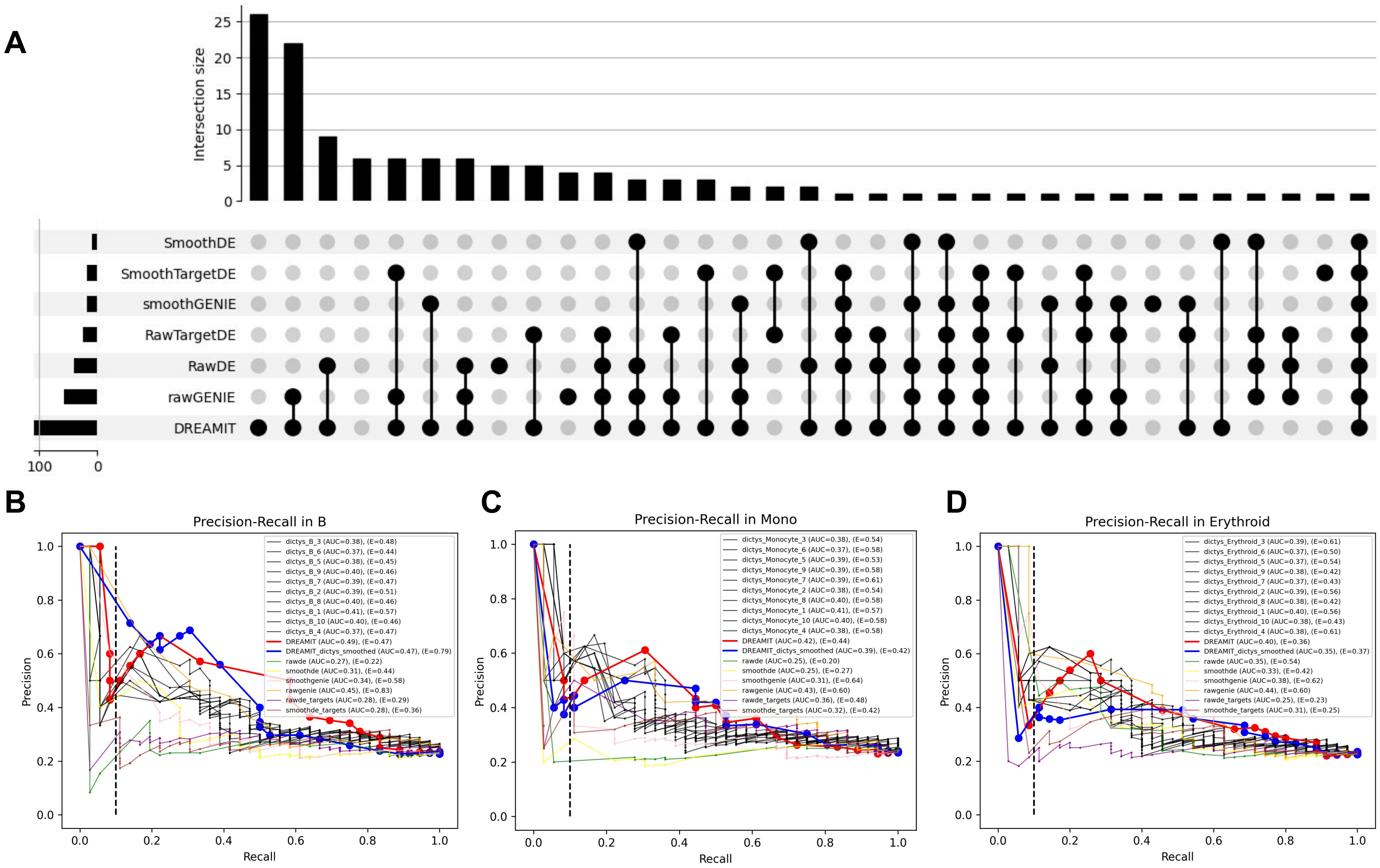

Fig S3. (A) Upset plot illustrating the set intersections between all methods of the TFs inferred for each branch; i.e. set members compared are TF-branch pairs. DREAMIT had the highest number of TF-branch pairs (109) with 26 uniquely predicted. DREAMIT and alternate methods' precision-recall of tissue specificity based on the TF-Marker database evaluated on three different branches. (B) B-cell lineage, (C) Monocyte lineage, and (D) Erythroid lineage. Dictys results are plotted in black, DREAMIT results using raw data and the DREAMIT spline smoothing approach are plotted in red, and DREAMIT results using pre-processed and smoothed data from Dictys are plotted in blue. Other methods, including Genie3, are also included (other colors, see legend). The early precision AUC is taken at 0.1 recall (dashed vertical line).

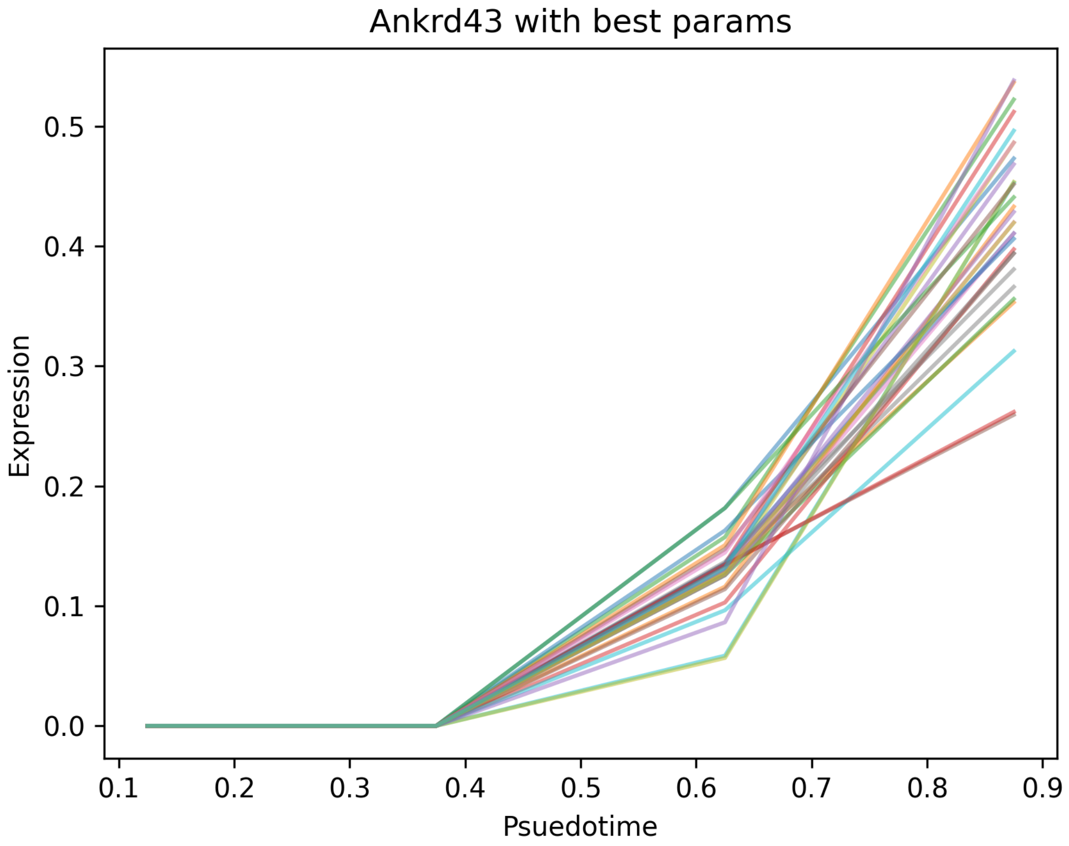

Fig S4. The 30 spline subsampled smooth data representations for Ankrd43 are plotted with expression plotted on the y-axis and pseudotime on the x-axis. This was using the best set of params (lowest AIC and CV) determined through the AIC and CV balancing depicted in Figure 5 and described in the methods.

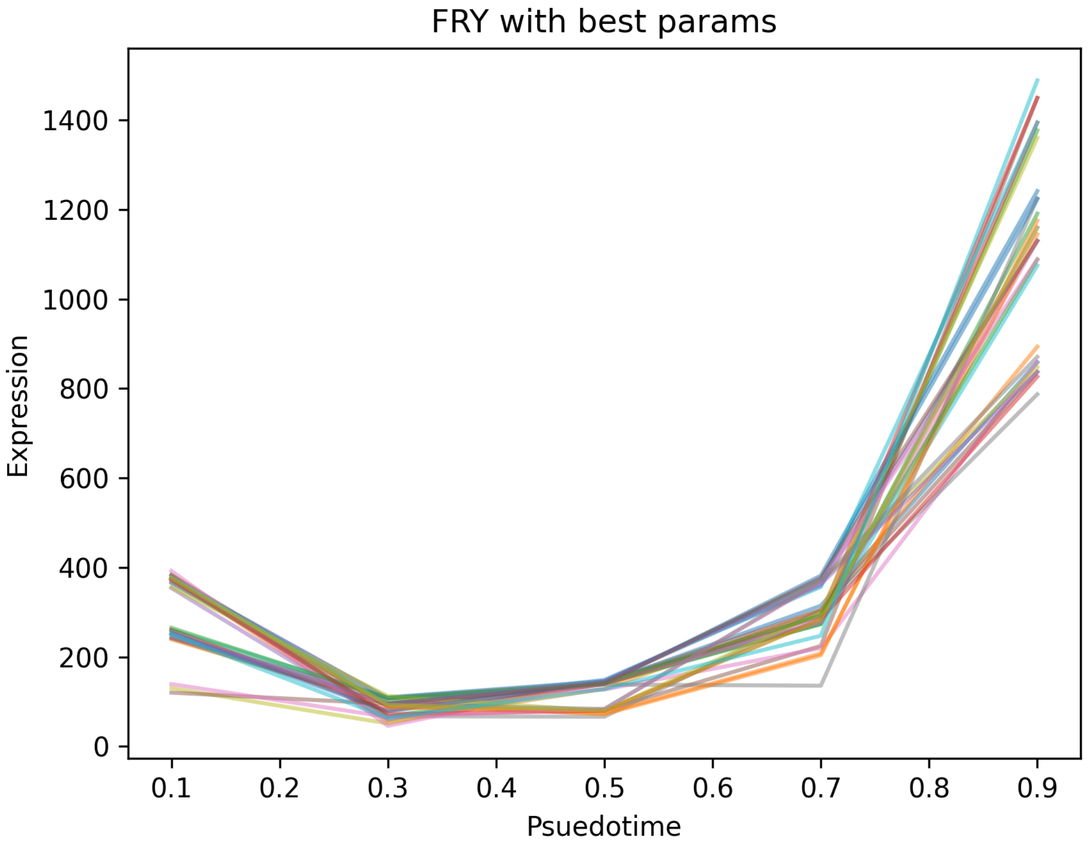

Fig S5. The 30 spline subsampled smooth data representations for FRY are plotted with expression plotted on the y-axis and pseudotime on the x-axis. This was using the best set of params (lowest AIC and CV) determined through the AIC and CV balancing depicted in Figure 5 and described in the methods.

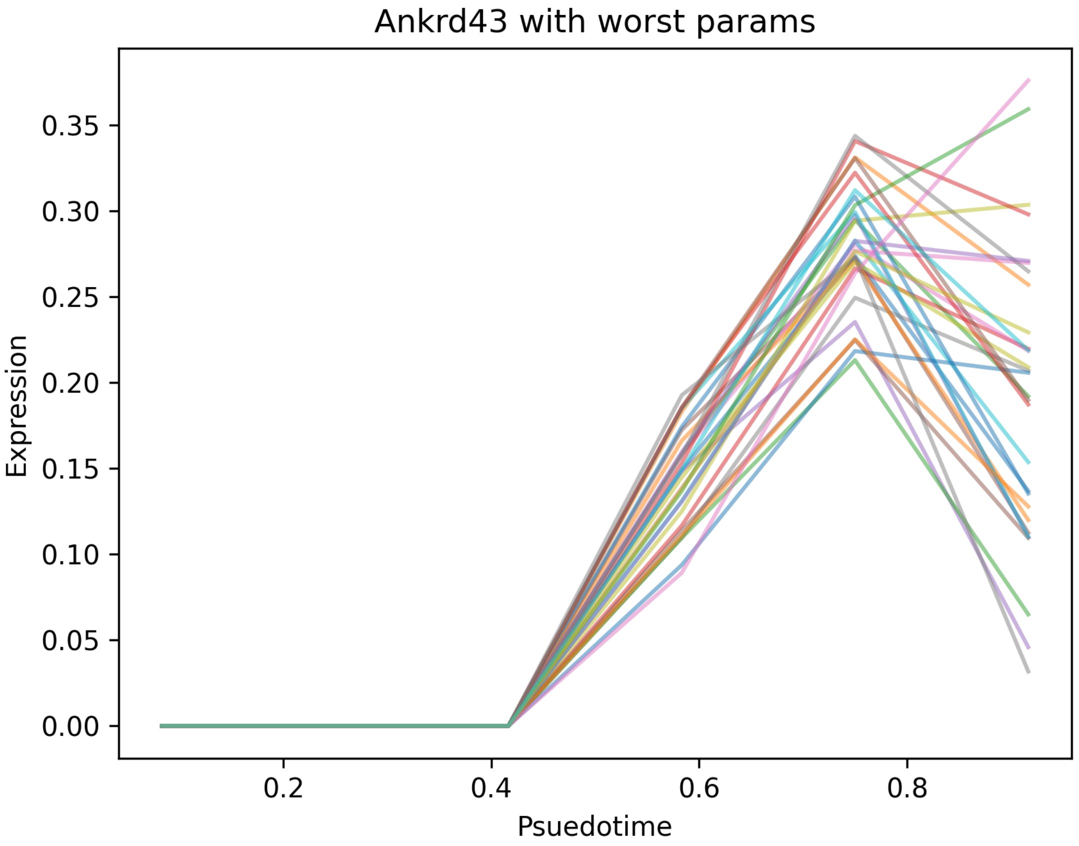

Fig S6. The 30 spline subsampled smooth data representations for Ankrd43 are plotted with expression plotted on the y-axis and pseudotime on the x-axis. This was using the worst set of params (highest AIC and CV) determined through the AIC and CV balancing depicted in Figure 5 and described in the methods.

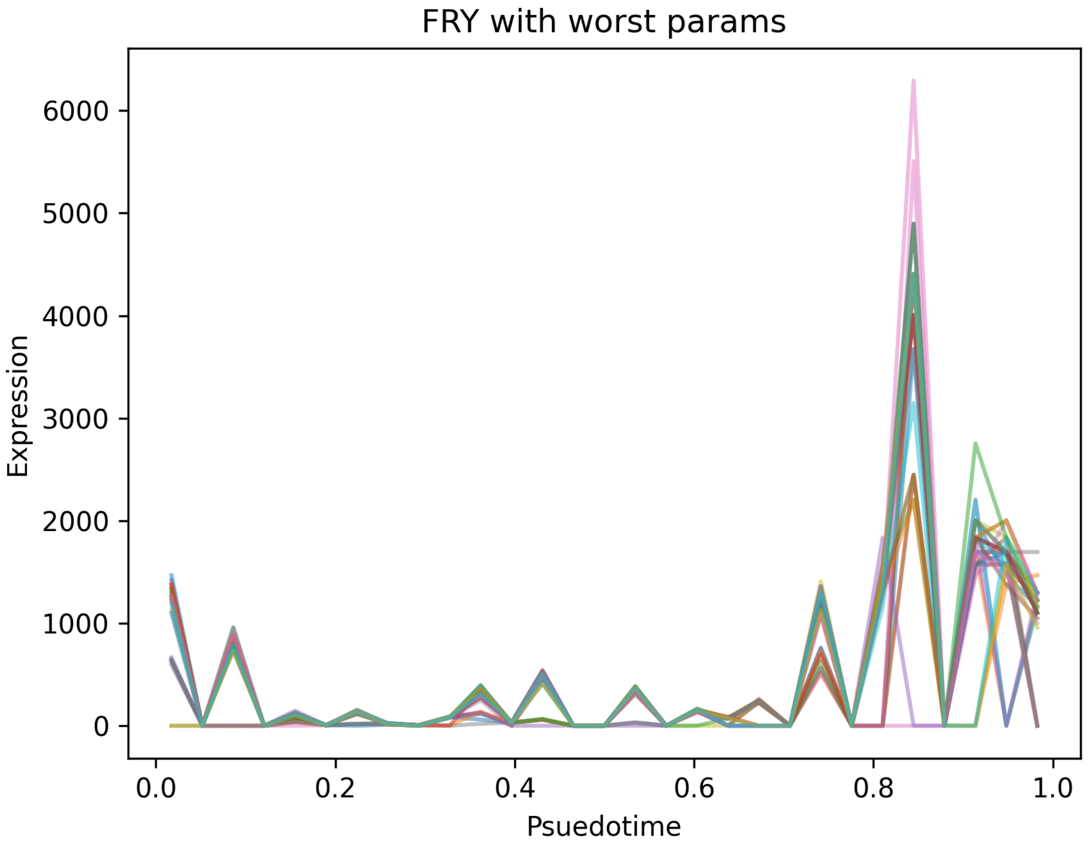

Fig S7. The 30 spline subsampled smooth data representations for FRY are plotted with expression plotted on the y-axis and pseudotime on the x-axis. This was using the worst set of params (highest AIC and CV) determined through the AIC and CV balancing depicted in Figure 5 and described in the methods.

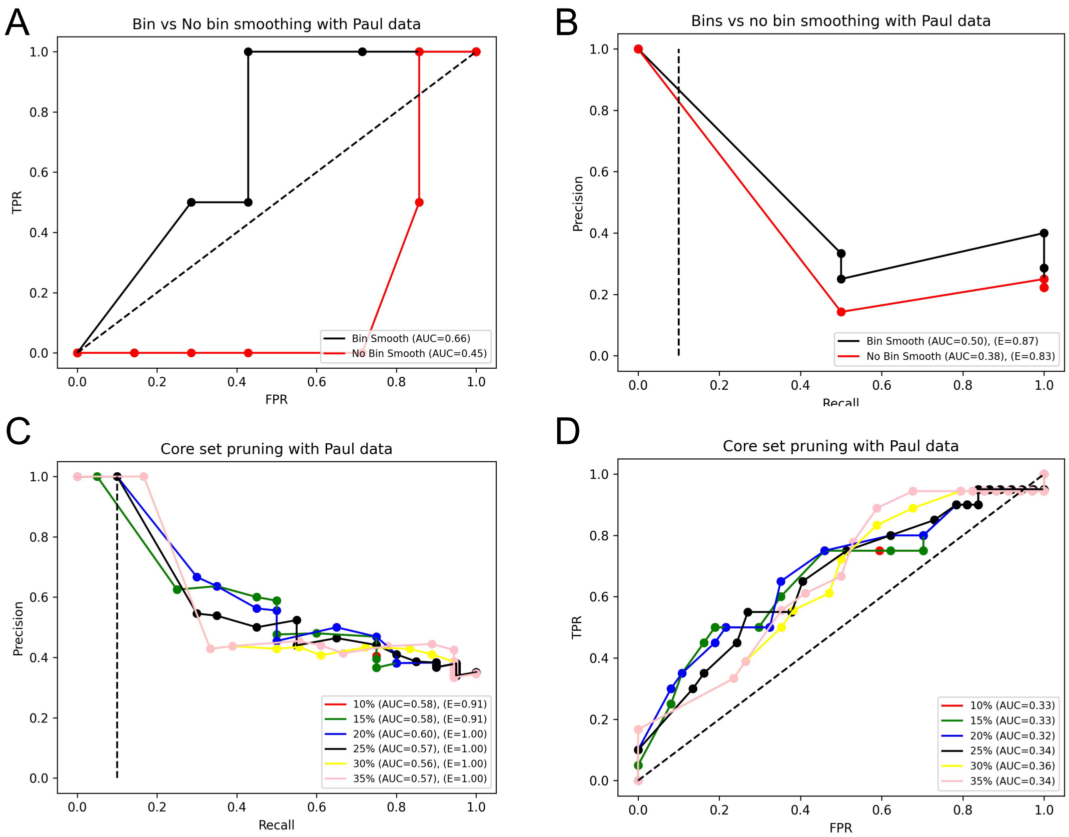

Fig S8. Using the same paul data plotted in Figure 2A the binning and pruning processes implemented by DREAMIT were compared to alternatives. Using binning for our spline smoothing (black) and spline smoothing without binning the raw data (red) is shown on a ROC plot (A) and precision-recall plot (B). Different thresholds for the pruning process were compared using precision-recall (C) and ROC (D). Early precision is plotted at a recall level of 0.1.
