# Supplementary material for: Associating transcription factors to single-cell trajectories with DREAMIT
Source: Genome Biol. 2024 Aug 14;25:220. doi: 10.1186/s13059-024-03368-7 (PMC11323358; doi:10.1186/s13059-024-03368-7)
Supplement: Supplementary file 3 — Additional file 3. Review history [file 13059_2024_3368_MOESM3_ESM.docx]

**1^st^ round**

**Reviewer 1**

In this manuscript, Maulding et al. provide DREAMIT (Dynamic Regulation of Across-Module Expression in Inferred Trajectories), a new framework for detecting patterns of TF activity along single-cell trajectory branching. Their method detects important TF target associations using a relation enrichment approach. Overall, this is an interesting method that appears to be superior to existing approaches for at least some applications, and will therefore be of interest to the community. However, some shortcomings need to be addressed.

1.There is not enough information about the runtime and scalability of this method. What is the running time of DREAMIT? How does the running time compare to other methods?
2.The dataset used in the article is single and lacks universality. I think the author should add the new dataset for comparison and analysis, so as to fully verify the performance of DREAMIT.
3.Most of the single-cell analysis methods developed now are based on deep learning or graph deep learning, such as scGNN[1], DESC[2], and scMGCA[3]. I think the author needs to add a comparison of these algorithms with DREAMIT and give the advantages of DREAMIT.
4.The authors demonstrate overall that DREAMIT provides better performance compared to other methods. However, in order for this approach to be truly interesting, they should show that DREAMIT can discover new biologically meaningful results that cannot be obtained by other methods.
5.Whether DREAMIT can be applied to data sets with time series information, such as Klein[4] and so on. I thought it would be interesting to be able to analyze the trajectory through time.
6.When calculating the pairwise association score between all targets and a specific TF, did the authors test other algorithms for calculating the association and whether it affected the results
7.I think the pictures in the article need to be normalized and further optimized.

[1]Wang J, Ma A, Chang Y, et al. scGNN is a novel graph neural network framework for single-cell RNA-Seq analyses[J]. Nature communications, 2021, 12(1): 1882.
[2]Li X, Wang K, Lyu Y, et al. Deep learning enables accurate clustering with batch effect removal in single-cell RNA-seq analysis[J]. Nature communications, 2020, 11(1): 2338.
[3]Yu Z, Su Y, Lu Y, et al. Topological identification and interpretation for single-cell gene regulation elucidation across multiple platforms using scMGCA[J]. Nature Communications, 2023, 14(1): 400.
[4]Klein A M, Mazutis L, Akartuna I, et al. Droplet barcoding for single-cell transcriptomics applied to embryonic stem cells[J]. Cell, 2015, 161(5): 1187-1201.

**Reviewer 2**

The article proposed a novel method, DREAMIT, which uses single-cell RNA sequencing data and locus inference methods to discover the dynamic regulatory role of transcription factors (tf) in cell state transition.This article evaluates DREAMIT's performance using a benchmark dataset containing multiple organizations and finds that it has higher tissue-specific sensitivity and specificity than other approaches. The authors also applied DREAMIT to the peripheral blood mononuclear cell dataset and discussed some TF networks associated with monocytes and red blood cells, revealing potential causal relationships between TFS.
However, I have some comments and suggestions :
The article did not fully demonstrate the content of the DREAMIT method, and lacked advantages of the method.The method proposed in this paper is not an algorithm, but only an analysis of the trajectory, not a new approach to find tf.The methods section of the article could have been clearer, such as using some flowcharts or pseudocode to show the main steps of the DREAMIT method; For example, use some formulas or examples to illustrate some key concepts and calculations involved in the DREAMIT method.There are some redundant contents in the method part, which need to be simplified to ensure that the contents are accurate and concise.
The background part of this paper can be more detailed, such as introducing the role and mechanism of transcription factors in cell state transition, as well as the current research progress and difficulties; For example, the advantages and disadvantages of existing methods in the analysis of TF dynamic regulation in single-cell trajectory data and their applicable scope are introduced. This will help readers better understand the importance and novelty of the questions raised by the article, as well as the advantages and innovations of the DREAMIT approach.
The English grammar of the article needs to be improved. It is suggested that the author proofread and polish it carefully to ensure that the language expression is clear and accurate.

**Reviewer 3**

DREAMIT is a computational algorithm to identify TFs that are predicted to have a key role in driving developmental trajectories using single-cell data. DREAMIT contains of the major steps: (1) Pseudotime ordering of cells is used to bin cells, (2) Average expression is computed in each bin, (3) Generalized Additive Models are fit using averaged expression to compute TF expression trends, (4) Predicted TF targets for each TF from the TRUSTT database are assessed for consistency with the TF expression trend using a number of different metrics, (5) A core set of targets is identified for each metric, (6) Distribution of scores for targets are assessed for significance using an empirical null, (7) TFs with significant concordance of target expression after multiple hypothesis correction. DREAMIT is applied to identify TFs in mouse hematopoiesis and outperforms the methods that are compared against.

Major concerns about the manuscript
1. My primary concern is a conceptual one - there have been a number of approaches recently to infer gene regulatory networks from single-cell data, particularly leveraging the multiomodal data (Reviewed in PMID: 37365273). I am not sure what is the degree of innovation and the conceptual leap-forward achieved by DREAMIT in relation to the gene regulatory network inference approaches. One recent approach, Dictys (PMID: 37537351) also discusses the dynamic nature of regulatory networks along differentiation trajectories.
2. Comments about the computational approach
a. The use of GAMs for estimating trends makes sense given the noise data. But in my opinion, the bin size used by authors is far too coarse and can mask a high degree of dynamics that are at play in differentiation trajectories. In fact, GAMs have been demonstrated to be sufficiently robust to fit reliable expression trends despite the strong density differences along pseudotimes (PMIDs: 30899105, 35027767).
b. The use of Kolmogorov-Smirnov tests as the primary measures of significance: KS tests are rather permissive and one of the major uses of tests in biology was to model the effect of miRNAs on their targets since the expression changes induced by miRNAs were so subtle. TFs have in general demonstrated to be a much stronger effect on gene expression and I am concerned that this test is not appropriate in this context.
c. 25th percentile of scores is used to define the core set of targets per TF - while a cutoff is understandable given the noise / uncertainty in TF-target predictions, the authors do not demonstrate the robustness of the results for this parameter
d. The authors mention in line 192 that 33 is a large number of targets - this is very surprising since TFs are known to regulate tens to hundreds of genes. What is the number of targets per gene ? How reliable are the results to changes in thresholds? And how consistent are the TF targets when context specific ATAC data is used?
3. Evaluation
a. The results on myeloid trajectory in 2B are interesting, but are such results obtained for other lineages too ?
b. Which specific metrics were used for evaluation in 2B? Is there literature evidence to suggest patterns of behavior consistent with these metrics
c. More detailed discussion of top TFs identified along with their ranking should be provided in addition to highlighting the TFs with literature evidence.
d. Comparison with single-cell gene regulatory network approaches should be presented to place DREAMIT in context.
e. Note that Paul et. al. is not a dataset of peripheral blood mononuclear cells but cells derived from bone marrow

**Authors’ response**

“Reviewer #1: In this manuscript, Maulding et al. provide DREAMIT (Dynamic Regulation of Across-Module Expression in Inferred Trajectories), a new framework for detecting patterns of TF activity along single-cell trajectory branching. Their method detects important TF target associations using a relation enrichment approach. Overall, this is an interesting method that appears to be superior to existing approaches for at least some applications, and will therefore be of interest to the community. However, some shortcomings need to be addressed.

“1.There is not enough information about the runtime and scalability of this method. What is the running time of DREAMIT? How does the running time compare to other methods?”

Response 1.1. We thank the reviewer for the comment about running time. Both DREAM-IT and Genie3 are much slower than the standard differential expression method. The longest part of the DREAM-IT method is the parameter grid search for spline fitting, which grows linearly with the number of cells along a trajectory (or in the dataset).

We here show the running time as a function of the dataset sizes for our study and obtained the following results:

EBI10 - 1207.8 S --- 57M

EBI4 - 2598.7 --- 239M

EBI5 - 46,273.3 --- 1.2G

EBI8 - 14.8 --- 52M

EBI9 - 537.0 --- 43M

friedman - 188,703.9 --- 174M

Paul_noregs - 5183.6 --- 188M

Paul_withregs - 5146.8 --- 188M

Parallelization can greatly speed this up as each grid search set of parameters is independent of another and so all can be run simultaneously on a larger compute cluster. We include a note on running time now in the Discussion to give readers an estimate of running time.

“2.The dataset used in the article is single and lacks universality. I think the author should add the new dataset for comparison and analysis, so as to fully verify the performance of DREAMIT.”

Response 1.2. We agree with the reviewer that the methods’ abilities should be demonstrated on multiple datasets. In our study, we now use 8 different datasets (7 in the original submission; 1 additional dataset for the Dictys comparison in response to referee #3’s comments; see Response 3.1) to illustrate the strengths and weaknesses of methods that cover a variety of data types and biological applications. We feel the selected datasets represent a breadth of different studies such as development, various tissues, and different temporal resolutions.

“3.Most of the single-cell analysis methods developed now are based on deep learning or graph deep learning, such as scGNN[1], DESC[2], and scMGCA[3]. I think the author needs to add a comparison of these algorithms with DREAMIT and give the advantages of DREAMIT.”

Response 1.3. We agree with the author that comparison to modern approaches is important. In fact, Reviewer 3 also asked us to compare to another method (Dictys), which we feel is more relevant than these deep-learning methods (please see Response 3.1). While deep-learning approaches are showing great recent progress, they still have not been widely adopted by the research community due to the resources required to train models and the resulting models are either not available or their are technical difficulties encountered when attempting to apply them off the shelf (e.g. due to batch effect issues with new platforms, etc). Even the most popular scVI is not used as much as it could be and why the now classic Genie3 method is still used by many researchers. Our goal was to also create a method that would be easily adopted by the research community and thus we do not feel comparing to DL methods is in scope.

The methods listed by the referee all project the cells into a lower dimensional manifold space in a different way, apply a trajectory inference method (Monocle in all cases) and then follow up with Differential Expression (DE) and other analyses. Thus, we view these transformation and trajectory methods as upstream approaches prior to running DREAMIT as DREAMIT can utilize any trajectory solution. We now make a note on this in the Discussion as well as provide a detailed comparison to the Dictys approach. Guided by this comment, we think it may be of interest to assess the dependence of our pipeline on the trajectory method (e.g. use Monocle instead of Slingshot). While the robustness analysis already included addresses this situation somewhat and we do include a result that uses a second trajectory method (PAGA instead of Slingshot), we agree using a different approach would be informative to assess whether DREAMIT’s performance is dependent on running Slingshot to achieve its level of performance. We now include a comparison with the competing method Dictys and use the trajectory input to its method (STREAM) instead of Slingshot to assess this dependence.

“4.The authors demonstrate overall that DREAMIT provides better performance compared to other methods. However, in order for this approach to be truly interesting, they should show that DREAMIT can discover new biologically meaningful results that cannot be obtained by other methods.”

Response 1.4. We agree with the referee and point out in the original text that we describe several examples that were found by DREAMIT and missed by other methods (lines 184 through 210 of original submission). The findings are summarized at a higher level in Figure 2B to point out the GRN “backbone” (TF to TF linkages) uncovered by DREAMIT. We believe the Results section is now more clear on this point as we have rewritten major portions of that part of the text to address referee #2 (see Response 2.3).

“5.Whether DREAMIT can be applied to data sets with time series information, such as Klein[4] and so on. I thought it would be interesting to be able to analyze the trajectory through time.”

Response 1.5. We agree that running on a time-series datasets would be highly useful. We now mention in the Conclusion as a potentially useful application of DREAMIT to future investigations and cite Klein et al as an example application dataset. In addition, the Dictys comparison we mention above in fact uses a time-oriented dataset and so these new results also begin to address this issue (see Response 3.1).

“6.When calculating the pairwise association score between all targets and a specific TF, did the authors test other algorithms for calculating the association and whether it affected the results.”

Response 1.6. We thank the referee for pointing out a possible point of confusion in our results and methodology. We do in fact use multiple metrics to quantify the association between TFs and their targets – Pearson correlation, rolling correlation, Spearman rank correlation, Mutual Information, and a dynamic time warping based metric (DTW). We have now made it more clear in the Background, Results, and Discussion that we use multiple metrics so that the reader is able to follow this aspect of our work more clearly.

“7.I think the pictures in the article need to be normalized and further optimized.”

Response 1.7. We apologize for any figure quality issues due to providing them in one manuscript document. For the revision, we now upload high-quality full resolution figure images as per the requirement of the final submission so that they can be reviewed more readily.

“Reviewer #2: The article proposed a novel method, DREAMIT, which uses single-cell RNA sequencing data and locus inference methods to discover the dynamic regulatory role of transcription factors (tf) in cell state transition.This article evaluates DREAMIT's performance using a benchmark dataset containing multiple organizations and finds that it has higher tissue-specific sensitivity and specificity than other approaches. The authors also applied DREAMIT to the peripheral blood mononuclear cell dataset and discussed some TF networks associated with monocytes and red blood cells, revealing potential causal relationships between TFS.

“However, I have some comments and suggestions :

“The article did not fully demonstrate the content of the DREAMIT method, and lacked advantages of the method.The method proposed in this paper is not an algorithm, but only an analysis of the trajectory, not a new approach to find tf. The methods section of the article could have been clearer, such as using some flowcharts or pseudocode to show the main steps of the DREAMIT method; For example, use some formulas or examples to illustrate some key concepts and calculations involved in the DREAMIT method. There are some redundant contents in the method part, which need to be simplified to ensure that the contents are accurate and concise.”

Response 2.1. We thank the reviewer for the suggestion to make the methods more clear. We now include a step-by-step overview at the top of the Methods section to orient the reader to the key ideas in the DREAMIT approach. We do discuss the advantages of the method over competing approaches (like differential expression and Genie3) and include a couple of notes on its limitations (e.g. considering only one TF at a time instead of multiple). We now include a running time limitation mention in response to this comment and Reviewer #1 (see Response 1.1 and also Response 1.3 for ideas on where DREAMIT fits in and relates to other single-cell approaches).

“The background part of this paper can be more detailed, such as introducing the role and mechanism of transcription factors in cell state transition, as well as the current research progress and difficulties; For example, the advantages and disadvantages of existing methods in the analysis of TF dynamic regulation in single-cell trajectory data and their applicable scope are introduced. This will help readers better understand the importance and novelty of the questions raised by the article, as well as the advantages and innovations of the DREAMIT approach.”

Response 2.2. We thank the referee for pointing out a relevant background topic to include. We have now included a paragraph in the Background section and have greatly revised the overall section. We now discuss TF regulation, the challenges of inference from RNAseq and scRNAseq, current approaches, and the current questions raised by these articles and approaches to motivate DREAMIT. One of the major additions in this version now includes a more specific framing of the problem statement in the context of this work and the particular solution provided by DREAMIT. We now make an argument that DREAMIT is one of the few tools for annotating TFs to specific cellular contexts within single-cell data like trajectory branches. We provide an analogy of inferring a GRN from (classic, bulk) RNAseq data versus inferring regulator activity in that data given an assumed GRN. Methods exist to infer GRNs from (current) single- cell RNAseq but few exist to infer activities of the regulators in the GRNs. This is what our approach tries to address. Again, we thank the referee for helping us better frame the work.

“The English grammar of the article needs to be improved. It is suggested that the author proofread and polish it carefully to ensure that the language expression is clear and accurate.”

Response 2.3. We have gone through the entire manuscript and improved the grammar and phrasing where we saw it was needed. Any changes in a red font, and without a comment stating otherwise, have been included to address this comment.

“Reviewer #3: DREAMIT is a computational algorithm to identify TFs that are predicted to have a key role in driving developmental trajectories using single-cell data. DREAMIT contains of the major steps: (1) Pseudotime ordering of cells is used to bin cells, (2) Average expression is computed in each bin, (3) Generalized Additive Models are fit using averaged expression to compute TF expression trends, (4) Predicted TF targets for each TF from the TRUSTT database are assessed for consistency with the TF expression trend using a number of different metrics, (5) A core set of targets is identified for each metric, (6) Distribution of scores for targets are assessed for significance using an empirical null, (7) TFs with significant concordance of target expression after multiple hypothesis correction. DREAMIT is applied to identify TFs in mouse hematopoiesis and outperforms the methods that are compared against.

“Major concerns about the manuscript

1. My primary concern is a conceptual one - there have been a number of approaches recently to infer gene regulatory networks from single-cell data, particularly leveraging the multimodal data (Reviewed in PMID: 37365273). I am not sure what is the degree of innovation and the conceptual leap-forward achieved by DREAMIT in relation to the gene regulatory network inference approaches. One recent approach, Dictys (PMID: 37537351) also discusses the dynamic nature of regulatory networks along differentiation trajectories.”

Response 3.1. We thank the referee for the comment. We first note that DREAMIT is not a GRN inference method per se, but instead infers relevant TFs to a trajectory branch given a known GRN. Please see our response to referee #2 (Response 2.2) as we have now made DREAMIT’s contribution more clear in the Background section. Having said that, we agree with the referee that comparison to other approaches would be helpful. Dictys also infers regulator activity through a procedure that aligns ATACseq and RNAseq data together followed by a probabilistic analysis. Thus, it makes sense to compare our approach with Dictys as both provide TF activity estimates. We do indeed find DREAMIT is comparable and sometimes more precising than Dictys even though it uses only the scRNAseq and ignores the scATACseq that Dictys uses (see new Supp Fig. 2). We have added a note in the Discussion section describing this motivation and comparison. As an aside, methods like Dictys (or another multi-omic approach) could use DREAMIT as a plugin for the step that generates a TF score from the RNAseq data (i.e. use DREAMIT’s association-based strategy).

“2. Comments about the computational approach

“a. The use of GAMs for estimating trends makes sense given the noise data. But in my opinion, the bin size used by authors is far too coarse and can mask a high degree of dynamics that are at play in differentiation trajectories. In fact, GAMs have been demonstrated to be sufficiently robust to fit reliable expression trends despite the strong density differences along pseudotimes (PMIDs: 30899105, 35027767).”

Response 3.2a. We thank the reviewer for the suggestion. To address this, we tried an approach that skipped the binning step and found that binning gives better specificity. We assume this is due to the presence of many zero-level expressed genes in the cells along any one branch. We now include these findings in the Methods section (as the results are quite technical in nature) where we now reference the new supplemental figure (Supp Fig. 7A-B). We note this result is likely dataset dependent as it will depend on the resolution (distance between cell transitions assayed). We provide the option to use either binning or no binning to obtain predictions with either approach.

“b. The use of Kolmogorov-Smirnov tests as the primary measures of significance: KS tests are rather permissive and one of the major uses of tests in biology was to model the effect of miRNAs on their targets since the expression changes induced by miRNAs were so subtle. TFs have in general demonstrated to be a much stronger effect on gene expression and I am concerned that this test is not appropriate in this context.”

Response 3.2b. We understand the caution expressed by the reviewer, but respectfully rebut the statement given ours and others' experience using the test on single-cell data. The KS test has been used in many different applications in RNAseq and scRNAseq analysis, popularized first for investigating genetic pathway enrichment in gene’s scored using t-tests on DNA microarray data (Subramanian et al. 2005). Many publications still make use of it when parametric assumptions about the score distributions between the observed and null can’t be made. In fact, our lab published a derivative of the test in 2019 called Biological Pathway Activity analysis for the specific application of an enrichment test even when using a single cell’s data (Ding et al. 2019). In this current study, we use binned pseudobulk data and therefore expect the use of KS to be even better behaved in this context compared to that work from several years ago. Perhaps the relevance of KS to single-cell analysis in this context reflects the ongoing subtlety of implicating a TF to its target set in the presence of the inherent noise of scRNAseq data

“c. 25th percentile of scores is used to define the core set of targets per TF - while a cutoff is understandable given the noise / uncertainty in TF-target predictions, the authors do not demonstrate the robustness of the results for this parameter.”

Response 3.2c. We thank the reviewer for the comment and have now tested different cutoffs (from 10% up to 35% in increments of 5%) to investigate how our original choice of 25% compares to other choices. Higher percentiles risk dropping out too many targets and many TFs have few to begin with in the database. Lower percentiles will not benefit from the noise reduction provided by removing targets with weak or no association to the factor. To gauge the effect, we used the Paul et al. dataset as an indicator of robustness. We found that 20 and 25% both produced comparable performance with better early precision than the other choices and so kept our original choice of 25%. We include this analysis in the Methods section and cite a new supplemental figure (Suppl Fig. 7C-D).

“d. The authors mention in line 192 that 33 is a large number of targets - this is very surprising since TFs are known to regulate tens to hundreds of genes. What is the number of targets per gene ? How reliable are the results to changes in thresholds? And how consistent are the TF targets when context specific ATAC data is used?”

Response 3.2d. We thank the referee for catching a typo in our original report. Instead of 33 targets, the number should have read 120. For TP53, the TRRUST database lists 164 target genes, of which 120 pass the scanpy variance filtering step and these are used in the TF-to-target association metric calculation step of DREAMIT. For the Pearson metric mentioned in the text, 78 targets were retained from the 120 as these also passed the RSEA target focusing step. We have now corrected this error.

“3. Evaluation

“a. The results on myeloid trajectory in 2B are interesting, but are such results obtained for other lineages too ?”

Response 3.3a. We do have results on other lineages – while no TFs were found to be significant in the “neuro” branch, a substantial and complex GRN that interlinks the TFs found to be significant was identified for the erythrocyte branch. We now include a GRN visualization of the implicated TFs for the stem to erythrocyte lineage (see new Supp Fig 1) and supporting literature evidence (see new Supp Table 1). Several interesting observations are present in the stem cell to erythrocyte lineage. RUNX1, EGR1, MYCN, and ETS1 increased in the early stages of the trajectory branch with widespread increased expression across the TF network in later stages. GATA1 and DNMT1, both regulated by a single distinct TF, show increases in expression following increases in their single regulator (MYC -> GATA1, DNMT1 -> TP53).

“b. Which specific metrics were used for evaluation in 2B? Is there literature evidence to suggest patterns of behavior consistent with these metrics.”

Response 3.3b. Figure 2B provides a visual GRN overview of DREAMIT’s findings for a trajectory branch, showing both which TFs are uncovered and linking them together if one appears in the target list of the other according to the TRRUST database. We hope the rewrite of the results makes this more clear now. The literature support for these are now provided in Supp Table 1.

“c. More detailed discussion of top TFs identified along with their ranking should be provided in addition to highlighting the TFs with literature evidence.”

Response 3.3c. We thank the reviewer and now include more TF examples for each of our main results. We added a new supplementary table (Supp Table 1) listing the top 20 TFs and added literature support. We now cite the table in the Results to make it clear which of the top TFs have biological relevance.

“d. Comparison with single-cell gene regulatory network approaches should be presented to place DREAMIT in context.”

Response 3.3d. We thank the reviewer for the suggestion. In this revision, we now explain more precisely that DREAMIT is not a GRN inference tool on its own. Instead, given a GRN, it attempts to infer which particular TFs are active for specific transitions in a single cell dataset. This distinction is made more clear in the Background section. Having said this, we did originally compare the method to Differential Expression (DE) and GENIE3 and have now added the comparison to the recently published method, Dictys, which also associates specific factors to trajectory branches and is comparable to DREAMIT (see Response X.X).

“e. Note that Paul et. al. is not a dataset of peripheral blood mononuclear

cells but cells derived from bone marrow.”

Response 3.3e. We thank the reviewer for correcting this error and have updated the manuscript.

**2^nd^ round**

**Reviewer 1**

The authors have addressed all my comments.

**Reviewer 2**

The revised manuscript now includes additional details that serve to elucidate the contents of this study. The primary objective of this investigation is to pinpoint the regulators responsible for "controlling cell transitions between and along trajectory branches" utilizing a newly devised framework known as DREAMIT (Dynamic Regulation of Expression Across Modules in Inferred Trajectories). However, following a comprehensive review of the revised manuscript, I would like to express the following concerns:

1.The primary concern of this study centers on the reliability of DREAMIT. To assess the performance of DREAMIT, the study employed a "bronze standard" to gauge its ability to recover transcription factors (TFs) known to be relevant to several datasets. However, the study's overarching objective is to identify regulators responsible for "controlling cell transitions between and along trajectory branches." Consequently, the focus should be on demonstrating the specificity of TFs along the trajectory rather than merely emphasizing the quantity of TFs. Even with the utilization of the "bronze standard," the performance of DREAMIT lacks persuasiveness. In the results pertaining to the prediction of PBMC markers, DREAMIT achieved an AUC of 0.57, a marginally superior performance to random guessing. Furthermore, in the context of identifying tissue-relevant TFs, although DREAMIT produced the best predictions, the AUC, at a mere 0.2, signifies predictions of little meaningful value.

2.Furthermore, the author conducted a comparison between DREAMIT and methods such as smoothGENIE, rawGENIE, smoothDE, rawDEtargets, and smoothDEtargets. It's worth noting that these methods were not originally designed to identify transcription factors enriched along pseudotime within a trajectory. The author acknowledges this limitation and mentions in the discussion that "Previous methods either ignore gene regulation, such as TRADE-seq or PseudotimeDE [17], or were not designed specifically for single-cell trajectories, such as GENIE3 [23]." Consequently, the comparative analysis between DREAMIT and these methods does not sufficiently substantiate the superiority and reliability of DREAMIT.

3.Another significant issue in this study pertains to the author's reliance on gene expression levels to establish relationships between transcription factors (TFs) and their target genes. It's crucial to recognize that the connection between them is primarily based on the binding motifs present on TF proteins and the DNA sequences of target genes. Expression levels alone cannot adequately represent the strength of the connections between TFs and their targets. Consequently, using Pearson and Spearman correlations based on gene expression levels is an incorrect approach for assessing the relationships between TFs and target genes. Similarly, relying on the similarity in expression patterns along pseudotime cannot provide substantial support for inferring relationships between TFs and their target genes for the same reasons.

4.The design of DREAMIT requires improvement. In this study, "slingshot and PAGA were used to infer trajectories and pseudotime," and the author subsequently acknowledged that "Errors introduced by a trajectory method can impact which gene regulatory relationships can be identified. DREAMIT is downstream of trajectory methods and is therefore subject to the same errors." In this context, DREAMIT cannot independently identify the regulators responsible for "controlling cell transitions between and along trajectory branches." If the results generated by DREAMIT were indeed reliable, the identified TF-target relationships should be employed as biomarkers to delineate cell states and trajectories.

In addition to the major concerns mentioned above, there are also several minor questions as follows:

5.In the main text: “For example, the factors STAT3 and RUNX1 are upregulated at the initial time point and their targets are upregulated in a later time point, consistent with the known regulatory relationships.” Is there any supporting reference?

6.What does "finds true biology" mean in this context? This study appears to offer limited biological evidence to validate the findings produced by DREAMIT.

7.“9 of them had established or emerging roles in stem and PBMC development in the literature.” Which literature?

8.What is the criteria of “more reliable” in the sentence of “The number of cells in a bin is used as a "weight" for the fitted data point, placing more emphasis on areas with more support that are assumed to have more reliable estimates of average expression” ?

9.It is hard to understand the following sentences: “Mutual Information (MI) describes how much factor expression informs (or could be used to predict) the expression of a target gene. This can be useful for trajectory analysis because it quantifies the information transmission between sender (factor) and receiver (target).”

10.What is the criteria of “the strongest relations” in the sentence of “DREAMIT performs a focusing step to retain a set of targets that mutually have the strongest relations to the TF.”

The investigation into the specificity of transcription factors (TFs) along the pseudotime of cell trajectories is undoubtedly intriguing. Nevertheless, there are fundamental issues in the design of DREAMIT, and the outcomes of this study do not adequately substantiate the reliability of DREAMIT.

**Reviewer 3**

I appreciate the responses of the authors. However, I continue to use doubts about robustness, scalability and usability of the manuscript in consideration of publication at Genome Biology.

1. The main issue continues to be that the presented work is more of a pipeline and not a formal statistical/ computational framework. As a result, formal methods for convergence etc for the overall solution to the problem are not available making the evaluation hard.

2. The authors point about binning in GAMs and use of KS-tests is well noted. But the permissiveness and/or sensitivity of these approaches mean true biological tests are necessary for validation of the method. I recognize that undertaking experiments to evaluate predictions are way beyond the scope of the study but the authors should have leveraged recent publications with large-scale perturbations of TF networks (Eg: https://pubmed.ncbi.nlm.nih.gov/37580596/ ) for evaluation.

3. Hematopoiesis is an excellent choice of model system for transcriptional study. However the particular study chosen by the authors is rather limited and has since been superseded by much larger-scale profiles in both human and mouse.

4. The computational scalability discussion added by the authors is welcome but I think it is far too limited which does not provide the use with sufficient information.

5. Similarly, the GitHub repository shared by the authors lacks detail in how a user can apply DREAMIT to their study.

**Authors’ response**

Reviewer #2: The revised manuscript now includes additional details that serve to elucidate the contents of this study. The primary objective of this investigation is to pinpoint the regulators responsible for "controlling cell transitions between and along trajectory branches" utilizing a newly devised framework known as DREAMIT (Dynamic Regulation of Expression Across Modules in Inferred Trajectories). However, following a comprehensive review of the revised manuscript, I would like to express the following concerns:

1.The primary concern of this study centers on the reliability of DREAMIT. To assess the performance of DREAMIT, the study employed a "bronze standard" to gauge its ability to recover transcription factors (TFs) known to be relevant to several datasets. However, the study's overarching objective is to identify regulators responsible for "controlling cell transitions between and along trajectory branches." Consequently, the focus should be on demonstrating the specificity of TFs along the trajectory rather than merely emphasizing the quantity of TFs. Even with the utilization of the "bronze standard," the performance of DREAMIT lacks persuasiveness. In the results pertaining to the prediction of PBMC markers, DREAMIT achieved an AUC of 0.57, a marginally superior performance to random guessing. Furthermore, in the context of identifying tissue-relevant TFs, although DREAMIT produced the best predictions, the AUC, at a mere 0.2, signifies predictions of little meaningful value.

Response 2.1. We agree with the reviewer that TF specificity, not just sensitivity, is extremely important. In fact, the results already demonstrate specificity of the TFs higher than random chance. In our case, a random AUC would not be 0.50 as our area is calculated on a curve of precision versus recall. The precision of random guessing across all recall levels would equal the fraction of tissue-relevant TFs out of the total. This value is also equal to the precision at the 100% recall level, which in our case is 35%. Thus, DREAMIT improves moderately on average over random guessing for this dataset when considering all recall levels, obtaining 57%. The closest point on the precision-recall curve to this average is at the 0.55 recall level, at which 0.55 precision is achieved and the difference is significant at the 0.05 level (P<0.026, hypergeometric test). Note also, as we point out in the paper, that DREAMIT achieves a much higher “early precision” (much higher precision than chance at lower recall levels). We have updated the manuscript to compare the performance more explicitly to chance guessing.

2.Furthermore, the author conducted a comparison between DREAMIT and methods such as smoothGENIE, rawGENIE, smoothDE, rawDEtargets, and smoothDEtargets. It's worth noting that these methods were not originally designed to identify transcription factors enriched along

pseudotime within a trajectory. The author acknowledges this limitation and mentions in the discussion that "Previous methods either ignore gene regulation, such as TRADE-seq or PseudotimeDE [17], or were not designed specifically for single-cell trajectories, such as GENIE3 [23]." Consequently, the comparative analysis between DREAMIT and these methods does not sufficiently substantiate the superiority and reliability of DREAMIT.

Response 2.2. In the manuscript revision, we received similar comments from the other two reviewers who asked that we expand our comparison to a method that was also specifically designed for the task of identifying TFs in trajectories. For this reason, we included a comparison to the suggested algorithm called Dictys as well as responses to that feedback (see responses 1.3 and 3.1 in the previous response to reviews) and note that both referees seem to be satisfied with this aspect of the study after the revision. In addition, we also now include a further demonstration of DREAMIT’s ability to identify relevant TFs by leveraging recently published PerturbSeq data as suggested by the third reviewer (see this response comment 3.2).

3.Another significant issue in this study pertains to the author's reliance on gene expression levels to establish relationships between transcription factors (TFs) and their target genes. It's crucial to recognize that the connection between them is primarily based on the binding motifs present on TF proteins and the DNA sequences of target genes. Expression levels alone cannot adequately represent the strength of the connections between TFs and their targets. Consequently, using Pearson and Spearman correlations based on gene expression levels is an incorrect approach for assessing the relationships between TFs and target genes. Similarly, relying on the similarity in expression patterns along pseudotime cannot provide substantial support for inferring relationships between TFs and their target genes for the same reasons.

Response 2.3. We appreciate the comment from the reviewer and agree with the concern that gene expression alone does not validate a TF to target connection. Since DREAMIT only uses a predefined set of TF-to-target connections (in this study provided by the TRRUST database), the starting set of relations already have documented evidence, independent of gene coexpression, that established the connection of a target with a factor (e.g. presence of a regulatory motifs or binding assays). DREAMIT should be viewed as a test of the relevance of a TF for a pseudotime branch much like a statistical gene set enrichment test could suggest that a pathway is implicated among an observed set of differentially expressed genes. In the case of differential expression, the differential expression alone does not prove a pathway is causally related to the experiment under study but suggests there is a putative connection that could also be due to indirect effects or common hidden causes.

We have added clarifying statements in the Introduction and Discussion sections to the manuscript to help a reader interpret DREAMIT’s results and to frame the specific concern raised here by the referee in the context of presenting the results.

4.The design of DREAMIT requires improvement. In this study, "slingshot and PAGA were used to infer trajectories and pseudotime," and the author subsequently acknowledged that "Errors introduced by a trajectory method can impact which gene regulatory relationships can be identified. DREAMIT is downstream of trajectory methods and is therefore subject to the same errors." In this context, DREAMIT cannot independently identify the regulators responsible for "controlling cell transitions between and along trajectory branches." If the results generated by DREAMIT were indeed reliable, the identified TF-target relationships should be employed as biomarkers to delineate cell states and trajectories.

Response 2.4. The referee’s comments suggest limitations due to factors outside the scope of improvements that can be made to the DREAMIT pipeline and statistical tests as presented and evaluated in the original and revised manuscripts. These factors include: a) the validity of the single-cell dataset, b) the trajectory inference method, and c) the catalog of known TF to target relations. We disagree with the referee that these sources of error suggest the design of DREAMIT is flawed, any more than other downstream analyses that rely on the results of upstream bioinformatics methods, are flawed.

Our method does account for poor data or poor results that could occur upstream of running DREAMIT. Namely, when splines are fit to trajectories, the method checks the coefficient of variation on the estimated parameters of the splines fit to 80% subsampled cells along the branch. This subsampling procedure was suggested by the authors of PseudotimeDE. To make it more clear, we have added statements in the Results and Discussion to make it obvious that poor quality data would not be considered for analysis.

In addition to the major concerns mentioned above, there are also several minor questions as follows:

5.In the main text: “For example, the factors STAT3 and RUNX1 are upregulated at the initial time point and their targets are upregulated in a later time point, consistent with the known regulatory relationships.” Is there any supporting reference?

Response 2.5. We thank the reviewer for pointing out this statement. We meant to suggest that the observed temporal order of upregulation in the dataset of these TFs relative to their target TFs (in the TF-to-TF network presented) was consistent with the network inferred by DREAMIT (not simply supported by the “known regulatory relationships” in TRRUST). We have rewritten this statement in the revised manuscript to make this clear and have provided citations to the supporting evidence of the TF-to-TF relationships that are relevant.

6.What does "finds true biology" mean in this context? This study appears to offer limited biological evidence to validate the findings produced by DREAMIT.

Response 2.6. We thank the referee for pointing out this vacuous statement. We have removed it and replaced it with a more appropriate summary of the results.

7.“9 of them had established or emerging roles in stem and PBMC development in the literature.” Which literature?

Response 2.7. The literature is tabulated in Supplemental Table 1 and we now make sure we cite this table for the literature evidence in the manuscript.

8.What is the criteria of “more reliable” in the sentence of “The number of cells in a bin is used as a "weight" for the fitted data point, placing more emphasis on areas with more support that are assumed to have more reliable estimates of average expression” ?

Response 2.8. The statement referred to here is a methodological statement that provides justification for putting more emphasis on areas of a pseudotime trajectory than *other areas of that same trajectory with fewer data points*, as data averages are more reliable when they are computed over more observations. We have added a qualifying statement to the manuscript to make this more clear.

9.It is hard to understand the following sentences: “Mutual Information (MI) describes how much factor expression informs (or could be used to predict) the expression of a target gene. This can be useful for trajectory analysis because it quantifies the information transmission between sender (factor) and receiver (target).”

Response 2.9. We have rewritten the description of mutual information to make it (hopefully) more clear to the reader.

10.What is the criteria of “the strongest relations” in the sentence of “DREAMIT performs a focusing step to retain a set of targets that mutually have the strongest relations to the TF.”

Response 2.10. The statement referred to by the referee is in the methods section where we describe how DREAMIT retains targets that have the highest scores to the TF based on the metric being considered (recall that DREAMIT uses several metrics). We rewrote this sentence to make sure the notion of “strongest relations” is clear. As far as what criteria is used to determine which targets are retained, the rest of the paragraph goes into detail on how a set of cutoffs were identified as those that maximized the resulting early precision cited in Supp Figure 7c-d.

Reviewer #3: I appreciate the responses of the authors. However, I continue to use doubts about robustness, scalability and usability of the manuscript in consideration of publication at Genome Biology.

1. The main issue continues to be that the presented work is more of a pipeline and not a formal statistical/ computational framework. As a result, formal methods for convergence etc for the overall solution to the problem are not available making the evaluation hard.

Response 3.1. The characterization of our approach as a pipeline versus an algorithm or a statistical model is indeed a nuanced aspect. It's worth noting that bioinformatics methods, ranging from pipelines to algorithms and conceptual frameworks, have all made significant contributions to the field. For example, Gene Set Enrichment Analysis published by Jill

Mesirov’s group could be considered a pipeline implementing the KS test, but it has clearly contributed substantially to bioinformatics analyses. Similarly, while DREAMIT integrates established metrics and a spline-smoothing GAM, it also introduces a Relational Set Enrichment Test to identify a core set of coregulated targets, thereby extending beyond a mere aggregation of existing methodologies.

2. The authors point about binning in GAMs and use of KS-tests is well noted. But the permissiveness and/or sensitivity of these approaches mean true biological tests are necessary for validation of the method. I recognize that undertaking experiments to evaluate predictions are way beyond the scope of the study but the authors should have leveraged recent publications with large-scale perturbations of TF networks (Eg:

https://pubmed.ncbi.nlm.nih.gov/37580596/ ) for evaluation.

Response 3.2. We appreciate the referee’s suggestion to compare our results to the recently published Lara-Astiaso et al. 2023 PerturbSeq comprehensive dataset performed in mouse, although we do note this study came out shortly before we submitted the revised manuscript. We collected the results of that study relevant for our hematopoietic lineages. Indeed, we found that the PerturbSeq results, collected in mice, were supportive of DREAMITs prediction in the human PBMC study that was performed. 8 of the TFs reported for the HSC to monocyte or HSC to HSC to erythrocyte were also associated with either of these branches in the human PBMC data. Even for the two TFs that were reported and not associated by DREAMIT, the levels of marker gene differential expression for the monocyte and erythrocyte branches were much lower presumably because these factors affect other parts of the hematopoietic system assayed in the mouse study. We now include this comparison in the manuscript and cite new Supplemental Figure 1 that has the results of the PerturbSeq as conformational evidence of DREAMIT annotations.

3. Hematopoiesis is an excellent choice of model system for transcriptional study. However the particular study chosen by the authors is rather limited and has since been superseded by much larger-scale profiles in both human and mouse.

Response 3.3. We agree with the referee and have expanded the evaluation with newer datasets. In addition to evaluating DREAMIT using the Lara-Astiaso dataset mentioned above, we incorporated the dataset used to compare to the Dictys method in the first round of reviews and as part of the first revision, which represents a larger-scale study in human hematopoiesis.

4. The computational scalability discussion added by the authors is welcome but I think it is far too limited which does not provide the [user] with sufficient information.

Response 3.4. The note on scalability referred to here in the Discussion was meant to provide a note on how speed increases could be obtained in the future by parallelizing the hyperparameter grid search (and thus reducing the user’s experienced “clock time”). The comment is meant for future developers to improve a user’s experience. We do now include information that is more helpful to a user in the GitHub repository (see next comment). For example, the number of threads used by the method can be set as a parameter to the DREAMIT code and this is illustrated in the GitHub example.

5. Similarly, the GitHub repository shared by the authors lacks detail in how a user can apply DREAMIT to their study.

Response 3.5. We have updated the GitHub repository to include a use case to give users a step-by-step guide on how to apply DREAMIT.

**3^rd^ round**

**Reviewer 2**

I appreciate the detailed response from the authors. In this version, the author has improved the wording to make the statement clearer to the reader. In addition, the comparison with the new method and the inclusion of recent data have improved the reliability of this study. Although, the DREAMIT method still exsit drawbacks in accuracy and sensitivity, according to the author, this study has made improvement compared to current methods. Therefore, I agree to accept the manuscript.

**Reviewer 3**

1. I would like to clarify that my comment regarding pipeline -vs- algorithm was strictly geared towards evaluation. I did not intend to downplay the efforts and ideas of the authors, and my apologies if it did. I continue to have concerns regarding evaluation.

2. It is encouraging that the the correct TFs were identified using the Perturb-seq data. But it is important to look at specificity too - are these TFs the most significant? Are other TFs identified at similar or greater significance? Is there biological meaning if such TFs exist?

3. The larger datasets was only use for comparison to the Dictys method and I am puzzled as to why the clearly richer datasets are not used for the assessments in the main results.

4-5. I disagree with the authors that the scalability issues should be reserved for a future development. The updated repository still does not provide any information about how a user can apply the tool to their own datasets. There is little documentation about inputs, formats, parameters and other critical elements.

**Authors’ response**

1. I would like to clarify that my comment regarding pipeline -vs- algorithm was strictly geared towards evaluation. I did not intend to downplay the efforts and ideas of the authors, and my apologies if it did. I continue to have concerns regarding evaluation.

Response 3.1. We thank the referee for the clarification and appreciate the additional ideas about evaluating the method.

2. It is encouraging that the correct TFs were identified using the Perturb-seq data. But it is important to look at specificity too - are these TFs the most significant? Are other TFs identified at similar or greater significance? Is there biological meaning if such TFs exist?

Response 3.2. We agree. We now include more description of the Perturb-Seq results to make it clear how they can be interpreted with respect to specificity. In summary, there were only eight TFs that could be tested by DREAMIT and that were also tested by Perturb-Seq. Perturb-Seq tested 81 TFs chosen to be relevant to hematopoiesis. Out of these, 37 overlapped with the TRRUST database. Out of these, 23 were retained after scanpy filtering on the PBMC dataset. Of these 23, 8 could be tested with DREAMIT (whereas 15 could not due to having target set sizes that were too small after eliminating those targets that also passed scanpy filtering).

Supp Figure 1 illustrates the results of all 8 of these TFs. Thus, the specificity estimate in this situation would be 2 out of 2 since the TFs identified as non-relevant indeed had lower perturbation scores across all of the lineages. However, the sample size is too low to construct any sort of confidence interval for specificity and so we are forced to report the findings as an anecdotal observation on the consistency between the DREAMIT and the Perturb-Seq results.

We’ve now added some more detail about the comparison to Perturb-Seq and an explanation of the small sample sizes of the TFs in common to help frame how to interpret the overlap with that study.

The relevant updates in the main text are:

* Results. First Section. Second Paragraph. First Sentence: “Eight TFs in common were either tested by Perturb-Seq (i.e. one of the 81 TFs chosen as relevant to the Lara-Astisao et al. hematopoietic study) and that had reliable target sets and data amenable for DREAMIT analysis on the PBMC data. E.g. 37 of the Perturb-Seq 81 overlapped with the TRRUST database and, out of these, 23 were retained after scanpy filtering on the PBMC dataset. 8 of these 23 could be tested with DREAMIT (whereas 15 could not due to having target set sizes that were too small after eliminating those targets that also passed scanpy filtering).”

* Results. Second Paragraph. Last Sentence: “The two TFs selected as non-relevant by DREAMIT indeed had lower Perturb-Seq scores across all of the lineages. While these results are consistent with DREAMIT identifying relevant TFs, the small number of TFs in common make impossible to calculate a quantitative level of specificity.”

3. The larger datasets [were] only [used] for comparison to the Dictys method and I am puzzled as to why the clearly richer datasets are not used for the assessments in the main results.

This is a good point. The reason for not including the Dictys results has more to do with the history of this manuscript’s review than anything else. The Dictys study was published after our paper was in review and we included a comparison to its results when advised to do so during the first round of reviews. The fact that the dataset is more comprehensive reflects the trend in molecular biology to advance technology for assessing higher numbers of cells as time goes by. Thus, our positioning of the results and comparison only to DREAMIT, rather than all of the alternate methods, was due to the “moving target” nature across our revisions. However, we agree with the referee that expanding our comparison to all of the other methods and reorganizing the results so that the Dictys comparison becomes one of the main benchmarks makes sense. To this end, we now compare the alternate methods, Genie3, Differential Expression (DE) and Dictys to DREAMIT on the Dictys dataset and have added additional figure panels to Figure 3 (see new Figure 3F for results summarizing precision-recall on all hematopoietic branches) and a supplemental figure (see Supp Figure 3B-D for precision-recall results on each of the separate branches). In summary, the results are mostly expected with the alternate methods scoring lower systematically compared to DREAMIT. However, Genie3 scores close to, or comparably, to DREAMIT (both have AUCs of 0.42), with Genie3 recording a slightly better early precision, while DREAMIT gains slightly higher precision in situations of higher recall. We have likewise updated the Results text to describe this expanded comparison.

The relevant changes in the manuscript are:

* Results. Third section. Last paragraph (and last paragraph in the entire Results section):

“To expand the evaluation of DREAMIT to a more comprehensive independent dataset, we compared the performance of DREAMIT and the other methods to a recent multi-omic study called Dictys [30], which used a probabilistic model to incorporate ATACseq and RNAseq for TF activity inference. All methods were compared based on their performance to Dictys on the Dictys’ human hematopoietic dataset analyzed by the Dictys authors with their published trajectory solution (using their STEAM method [31]). Surprisingly, we found that the performance of DREAMIT and Genie3 were comparable to Dictys (DREAMIT and Genie3 both achieved AUCs of 0.42, while Dictys achieved 0.41 overall), with Genie3 having slightly higher early precision and DREAMIT higher late precision (see Figure 3F). Notably, DREAMIT’s and Genie’’s precision was higher than Dictys over most recall levels considering all 10 different temporal GRNs produced by Dictys for each of the three trajectory branches. The alternate methods were not as competitive. On some of the branches, DREAMIT had superior performance. On the B-cell branch for example, DREAMIT had a better overall performance (0.49) compared to the best precision achieved by Dictys (0.41) or Genie (0.45) (see Additional file 1: Fig. S3B-D for a breakdown of results on all three hematopoietic branches). We found that using the Dictys smoothing of the data did not influence the performance of the methods.”

* Legend of new Figure 3F: “F.DREAMIT and alternate methods’ precision-recall of tissue specificity based on the TF-Marker database evaluated on, and aggregated over, three different branches – B-cell, Monocyte, and Erythroid lineages. Results for several methods were compared including: Dictys (10 black lines), DREAMIT using raw, unprocessed data and its own spline smoothing (red), DREAMIT using pre- processed and smoothed data from Dictys (blue), Genie3 using raw data (orange) and Dictys-smoothed processed data (pink), as well as other methods and preprocessing (see legend). The early precision AUC is taken at 0.1 recall (dashed vertical line).”

* Legend for new Supp Fig S3B-D: “DREAMIT and alternate methods’ precision-recall of tissue specificity based on the TF-Marker database evaluated on three different branches. (B) B-cell lineage, (C) Monocyte lineage, and (D) Erythroid lineage. Dictys results are plotted in black, DREAMIT results using raw data and the DREAMIT spline smoothing approach are plotted in red, and DREAMIT results using pre-processed and smoothed data from Dictys are plotted in blue. Other methods, including Genie3, are also included (other colors, see legend). The early precision AUC is taken at 0.1 recall (dashed vertical line).”

4-5. I disagree with the authors that the scalability issues should be reserved for a future development. The updated repository still does not provide any information about how a user can apply the tool to their own datasets. There is little documentation about inputs, formats, parameters and other critical elements.

Response 3.4. We thank the referee for the comment about scalability. Another critical element of running the method is how it can be parallelized, in addition to mentioning the expected running time using a certain number of threads as was previously included in the README, we now also include the architecture of the machine we obtained these estimates on to help interpret the timing we give. We also now repeat these typical expected time estimates in the Discussion section of the main text. In addition, we also describe the relevant parameters that can be used to adjust how the code is run in case a user has more/less CPUs at their disposal.

Response 3.5. We thank the referee for ideas about making the code more accessible. We have added some more clarification to the repos README.md markdown file, which is the assumed starting point that a user will look at if interested in running our method on their dataset. We include excerpts from the manuscript that walk a user through key overview figures of the method in the README.md file. In addition, we added a new TUTORIAL section that explicitly illustrates a working example and shows examples of using parameters and file formats. We now describe what the input datasets are and what their formats look like explicitly in the README file. This includes all of the inputs and all possible parameters that can be used with the code.

As provided in the main text, please see https://github.com/nathanmaulding/DREAMIT

for a major update to the repository.

Relevant changes to the manuscript for this response are:

* Discussion section last paragraph: “ A typical dataset, like the one taken from Paul et al. (Paul et al. 2015) and provided as an example in the code repository, takes one hour with 20 threads.”

* And the updated github repo: https://github.com/nathanmaulding/DREAMIT.git.
